# An Integrative Genomic Island Affects the Adaptations of the Piezophilic Hyperthermophilic Archaeon *Pyrococcus yayanosii* to High Temperature and High Hydrostatic Pressure

**DOI:** 10.3389/fmicb.2016.01927

**Published:** 2016-11-29

**Authors:** Zhen Li, Xuegong Li, Xiang Xiao, Jun Xu

**Affiliations:** ^1^State Key Laboratory of Microbial Metabolism, School of Life Sciences and Biotechnology, Shanghai Jiao Tong UniversityShanghai, China; ^2^Institute of Oceanology, Shanghai Jiao Tong UniversityShanghai, China; ^3^Deep-Sea Cellular Microbiology, Department of Deep-Sea Science, Sanya Institute of Deep-Sea Science and Engineering, Chinese Academy of SciencesSanya, China

**Keywords:** deep-sea, piezophilic hyperthermophile, *Pyrococcus*, genomic island, integrative element, adaptation

## Abstract

Deep-sea hydrothermal vent environments are characterized by high hydrostatic pressure and sharp temperature and chemical gradients. Horizontal gene transfer is thought to play an important role in the microbial adaptation to such an extreme environment. In this study, a 21.4-kb DNA fragment was identified as a genomic island, designated PYG1, in the genomic sequence of the piezophilic hyperthermophile *Pyrococcus yayanosii*. According to the sequence alignment and functional annotation, the genes in PYG1 could tentatively be divided into five modules, with functions related to mobility, DNA repair, metabolic processes and the toxin-antitoxin system. Integrase can mediate the site-specific integration and excision of PYG1 in the chromosome of *P. yayanosii* A1. Gene replacement of PYG1 with a *Sim*^R^ cassette was successful. The growth of the mutant strain ΔPYG1 was compared with its parent strain *P. yayanosii* A2 under various stress conditions, including different pH, salinity, temperature, and hydrostatic pressure. The ΔPYG1 mutant strain showed reduced growth when grown at 100°C, while the biomass of ΔPYG1 increased significantly when cultured at 80 MPa. Differential expression of the genes in module III of PYG1 was observed under different temperature and pressure conditions. This study demonstrates the first example of an archaeal integrative genomic island that could affect the adaptation of the hyperthermophilic piezophile *P. yayanosii* to high temperature and high hydrostatic pressure.

## Introduction

Deep-sea hydrothermal vent environments are characterized by high hydrostatic pressure (HHP) and sharp temperature and chemical gradients (Reysenbach et al., 2006; Oger and Jebbar, 2010). The microorganisms dwelling here are expected to show strong high temperature adaptation (Jebbar et al., 2015). Mobile genetic elements, such as plasmids, bacteriophages, transposons, integrons, conjugative transposons, integrative conjugative elements (ICEs), and genomic islands (GIs), are important and essential components of the marine biosphere that promote marine microbial diversification (Sobecky and Hazen, 2009). Horizontal gene transfer (HGT) of mobile genetic elements is assumed to play an important role in the microbial adaptation to extreme environments (van Wolferen et al., 2013).

Many of the accessory genes acquired by HGT form syntenic blocks recognized as GIs (Juhas et al., 2009). These gene fragments are often inserted into tRNA gene loci, which act as integration sites for foreign DNA, mainly prophages, and are flanked by direct repeats (DRs) that consist of a few to more than a hundred nucleotides. Many GIs can spontaneously excise from and integrate into the chromosome, while some of them can lose this mobility. Integrases, transposases, ISs, and other mobility genes encoded in GIs can be involved in the integration, mobility, deletion, and rearrangement of GIs (Darmon and Leach, 2014).

The GC content and the codon usage of GIs are generally different from the other regions of the chromosome. GIs are typically recognized as discrete DNA segments between closely related strains, and these elements might contribute to the diversification and adaptation of microorganisms, thereby significantly impacting genome plasticity and evolution (Polz et al., 2013). For instance, seven novel cell wall-associated GIs delineated two major clades within the halophilic archaeon *Haloquadratum walsbyi* genome, and this type of variation probably reflects a number of mechanisms that minimize the infection rate of viruses (Martin-Cuadrado et al., 2015). The transcriptome of the piezophile *Photobacterium profundum* SS9 grown under different pressure (28 MPa vs. 45 MPa) and temperature (4°C vs 16°C) conditions was analyzed, and the results showed that there were differentially expressed genes that belonged to three GIs (Chr1.8, Chr2.3, and Chr2.5) in the SS9 genome; these genes are absent in both the pressure-sensitive strain 3TCK and the pressure-adapted strain DSJ4 (Campanaro et al., 2005). In addition, genes that are responsible for the defense function, including the toxin-antitoxin system, restriction-modification system, phage abortive infection system, and CRISPR/Cas system, were frequently identified in GIs in various bacteria and archaea (Dobrindt et al., 2004; Makarova et al., 2011).

*Thermococcales* are widely distributed in geothermal environments, including hot springs, volcanoes, and deep-sea hydrothermal vents (Bertoldo and Antranikian, 2006; Stetter, 2006; Ferrera and Reysenbach, 2007). The order *Thermococcales* is represented by three genera, *Thermococcus, Pyrococcus*, and *Paleococcus*, which are obligate anaerobic heterotrophic hyperthermophiles (Bertoldo and Antranikian, 2006; Ferrera and Reysenbach, 2007). Genetic elements with the characteristics of GIs have also been identified in *Thermococcales*. Four virus-like regions (TKV1 through TKV4) have been found in the genome of *T. kodakarensis* (Fukui et al., 2005), and the genes in these virus-like integrated elements were found to be capable of stimulating cell growth at 85°C in nutrient-rich medium (Tagashira et al., 2013). Six putative highly variable GIs have been identified among the eight *Pyrococcus* genomes (White et al., 2008), suggesting that maintenance of the microbial phenotypic diversity by extensive genome rearrangements and HGT help to respond to rapidly changing environmental conditions.

*Pyrococcus yayanosii* CH1 is the first example of the strictly piezophilic hyperthermophilic archaeon isolated from the mid-Atlantic Ridge hydrothermal vents (4,100-m depth; Zeng et al., 2009; Birrien et al., 2011). A complete genome sequence of a 1.7-Mb circular chromosomal DNA molecule of the model strain *P. yayanosii* CH1 was announced (Jun et al., 2011), and a gene disruption system has been developed (Li et al., 2015). In the present study, a typical GI (PYG1) in the *P. yayanosii* genome was identified and genetically characterized. The mobility of PYG1 was confirmed, and an artificial GI was constructed to investigate the integration process. Moreover, growth differences between the parent strain and the PYG1 deletion mutant strain under high temperature and HHP suggested that this archaeal integrative GI plays an important role in the environmental adaptation of this species.

## Materials and Methods

### Strains and Plasmids, Media and Growth Conditions

The plasmids and strains used in the present study are listed in **Table 1**. *P. yayanosii* A1, a facultative piezophilic derivative strain of *P. yayanosii* CH1, was cultivated in 100 ml serum bottles under anaerobic conditions at 95°C and 0.1 MPa in 30 ml of TRM (Zeng et al., 2009; Li et al., 2015) containing 3.3 g PIPES disodium salt, 30 g NaCl, 5 g MgCl_2_ 6H_2_O, 0.7 g KCl, 0.5 g (NH_4_)_2_SO_4_, 1 ml KH_2_PO_4_ 5%, 1 ml K_2_HPO_4_ 5%, 1 ml CaCl_2_ 2H_2_O 2%, 0.05 g NaBr, 0.01 g SrCl_2_ 6H_2_O, 1 ml Na_2_WO_4_ 10 mM, 1 ml FeCl_3_ 25 mM, 1 g yeast extract, 4 g tryptone, and 1 mg resazurin. After transformation, the strains were selected on TRM supplemented with 10 μM simvastatin (Sigma). Gelrite (1.5% w/v) was added to solidify the medium. The medium pH was adjusted from 5.8 to 8.2 by adding 1 M HCl or 1 M NaOH. The salinity of the medium was adjusted by adding different amounts of NaCl. The growth was monitored by cell counting using a Thomas chamber and light microscopy at a magnification of ×40 (Zeng et al., 2009). The *Escherichia coli* strain DH5α was used for general DNA manipulation, and the *E. coli* was cultivated in Luria-Bertani (LB) medium at 37°C.

**Table 1:** Strains and plasmids used in the present study.

Strains and plasmids	Description	Reference
**Strains**		
*P. yayanosii* A1	Facultative piezophilic derivative strain.	Li et al., 2015
*P. yayanosii* A2	*pyrF* gene knockout strain of *P. yayanosii* A1.	Li et al., 2015
Δ*int*	A mutant strain that the integrase PYCH_15110 was replaced by a *Sim*^R^ cassette in *P. yayanosii* A2.	This study
ΔPYG1	Most of genes (PYCH_15120∼PYCH_15330, 19,691 bp) in PYG1 were replaced by a *Sim*^R^ cassette.	This study
**Plasmids**		
pLMO12102	pGT5 replication area of *P. abyssi* GE5 which contains *sso, dso*, and Rep75 protein were inserted in pUC18 plasmid of *E. coli.*	Lab stock
pLMO04	Derivative of pLMO03, without *Sim*^R^ cassette.	This study
pLMOZ1402	Z1402 was inserted in pLMO12102 at *Kpn* I site.	This study
pLMOZ1404	Z1404 was inserted in pLMO12102 at *Kpn* I site.	This study
pLMOZ1405	Z1405 was inserted in pLMO12102 at *Kpn* I site.	This study
**Artificial GIs**	DNA segments	
Z1402	Cloning and fusion two flanking sequences of PYG1 containing 1,263-bp *attL-int* and 917-bp PYCH_15340-tRNA^Gln^, *Sim*^R^ cassette was inserted at *Cal I* site.	This study
Z1404	Derivative of Z1402, without *attL.*	This study
Z1405	Derivative of on Z1402, without PYCH_15110 (*int*).	This study

### HHP Culturing Experiments

All manipulations before the pressurized culturing experiments were performed anaerobically inside an anaerobic glove box (Coy Lab). Cultivation of *P. yayanosii* was performed using a custom-built high pressure/high temperature incubation system similar to that reported by Zeng et al. (2009). A 10-ml plastic syringe was used as the container of liquid medium. After inoculation, the needle head of the syringe was sealed tightly with a butyl rubber stopper. The syringe was then placed inside a titanium chamber that was pressurized to the appropriate hydrostatic pressure (e.g., 52 MPa) and maintained at high temperature (95°C).

### Construction of a Series of Artificial GIs

The recombination plasmid pLMOZ1402 harboring the artificial GI Z1402 was constructed in accordance with the method described by He et al. (2007). The upstream arm containing the *att* site and the integrase PYCH_15110 (1263 bp, locus positioning from 1321406 to 1322668 in the genome) and the downstream arm containing PYCH_15340 and tRNA^Gln^ (917 bp, locus positioning from 1342360 to 1343276 in the genome) were obtained through PCR amplification. A fusion fragment comprising the upstream and downstream arms with *Kpn* I restriction enzyme sites at the extremities was constructed using overlap extension PCR. The overlapping region contained a *Cal* I restriction enzyme site. The fusion fragment was inserted into the pLMO12102 plasmid and digested using *Kpn* I, generating the intermediate plasmid pLMOZ140i. The 1508-bp *Sim*^R^ cassette, harboring *Cal* I recognition sites at the extremities, was amplified from pLMO03 using the primers *Sim*^R^-F/R (Supplementary Table S1). Subsequently, the *Sim*^R^ cassette was inserted into plasmid pLMOZ140i at the *Cla* I site. The resulting plasmid was referred to as pLMOZ1402. Recombination plasmids pLMOZ1404 (mini-island Z1404 without the *att* site) and pLMOZ1405 (mini-island Z1404 without the *int* gene) were also constructed in the same manner.

### Bioinformatics Analysis

The GI was identified in the genome sequence of *P. yayanosii* (GenBank accession No. NC_015680) using the web-based tool IslandViewer^1^ (Langille and Brinkman, 2009). The nucleotide sequences of GIs were analyzed using BLAST programs. The functions of putative ORFs were predicted through comparisons with sequences in GenBank using the BLASTP algorithm. Multiple DNA and amino acid sequence alignments were performed using ClustalW or DNAMAN 6.0. The phylogenetic analysis was performed using the neighbor-joining tree algorithm with a software package for constructing evolutionary trees (MEGA, version 6.0).

### Genetic Manipulation

At the end of the exponential phase, the genomic DNA from *P. yayanosii* A1 was extracted as previously reported (Li et al., 2015). The total RNA of *P. yayanosii* A1 was extracted by Trizol. RNA purification was performed with DNase I (Thermo). The cDNA was prepared using a cDNA synthesis kit (Thermo). The plasmid DNA was extracted from *E. coli* using the plasmid extraction kit (Omega). The DNA purification was conducted using a DNA gel extraction kit or the Cycle-Pure Kit (Omega). The restriction endonucleases and T4 ligase were purchased from Takara or NEB. DNA sequencing and oligonucleotide synthesis were performed at Sangon (Sangon Biotech).

*Pyrococcus yayanosii* A1 was transformed according to the method described by Li et al. (2015). *P. yayanosii* A1 was cultivated in 50 ml of TRM at 95°C and 0.1 MPa for 12 h, and cells in the late exponential growth phase were harvested and subsequently resuspended in 200 μL of cold transformation buffer (80 mM CaCl_2_). The suspended cells were incubated on ice for 0.5 h under anaerobic conditions, and subsequently 3 μg of DNA was added to the suspension and incubated on ice for 1 h. After a heat shock at 95°C for 45 s, the suspension was incubated on ice for 10 min. The treated suspension was transferred to 8 ml of TRM (without simvastatin) and cultured for two generations. The culture was spread onto solid TRM supplemented with 10 μM simvastatin and further incubated for 3–5 days at 95°C.

### PCR and Real-Time PCR Conditions

The primers used in the present study are listed in Supplementary Table S1. The following PCR cycling conditions were used: high temperature pre-denaturation at 95°C for 5 min, followed by 30 cycles of denaturation at 94°C for 30 s, annealing at a primer-specific temperature for 30 s, and a final extension at 72°C for a duration dependent on the length of the expected amplification products.

Real-time PCR was performed using an Applied Biosystems 7500 Real-Time PCR System and Power SYBR^®^ Green PCR Master Mix (Applied Biosystems). The 16S rRNA gene was used as reference gene and amplified with primers P17 and P18 to detect the rate of PYG1 circularization. The following real-time PCR conditions were used: 40 cycles of denaturation at 95°C for 15 s, annealing at 54°C for 30 s, and extension at 65°C for 1 min, followed by a final cycle at 95°C for 15 s, 60°C for 1 min, 95°C for 30 s, and 60°C for 15 s. The copy number for the reference gene 16S rRNA was assigned a value of 100%, and the rates of circularization were presented as a calculated percentage relative to the copy numbers of the reference gene.

## Results

### Identification of GIs in *P. yayanosii*

**FIGURE 1:**
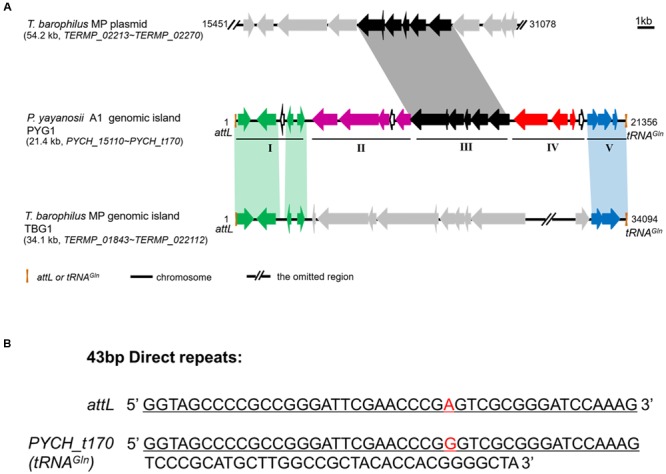
**Structural characteristics of PYG1 showed a close relationship between PYG1, TBG1, and a plasmid of *T. barophilus* MP. (A)** Summary of PYG1 features. Organizational map of the 23 predicted open reading frames (ORFs) on the chromosomally integrated PYG1 element extending from the left junction *attL* (Orange bar) to the right junction tRNA^Gln^ (Orange bar). The omitted regions of the TBG1 or plasmid sequence were replaced by double slash (//). The five modules (I–V) referred to in the text are shown as thin solid lines. In module I, the ORFs in green are related to the integration of PYG1 and shared 82–98% identity to the ORFs in TGB1. In module II, the ORFs in purple are related to the McrBC 5-methycytosine-dependent restriction endonuclease and a methyladenine DNA glycosylase. The ORFs shown in black (module Δ) are predicted to be involved in metabolism and represent homologs of the *T. barophilus* MP plasmid. In module IV, the ORFs are shown in red, and PYCH_15290 encodes a putative transposase. The ORFs shown as hollow black arrows in module V also exhibited homology with TBG1. There are three ORFs in dark blue that do not show any homology with proteins in the GenBank databases. The gray arrows mean that these ORFs have no homology with PYG1. **(B)** Sequence of the 43-bp direct repeats indicated in *attL* and PYCH_t170.

Using IslandViewer, 15 putative GIs were identified in the genomic sequence of *P. yayanosii* CH1 (**Table 2**). The largest GI, a 21,356-bp GI ranging from 1321661 to 1343016 in the chromosome, was named PYG1 for further characterization (Supplementary Figure S1). The boundaries of PYG1 were defined by DRs of 43 bp and the 3′ terminus of the tRNA^Gln^ gene (PYCH_t170; **Figures 1A,B**). The GC content in PYG1 (41.3%) is lower than the average GC content (51%) of the *P. yayanosii* genome. Another 17,552-bp GI, named PYG2, ranging from 1238311 to 1255863, was found to be integrated into the 3′ terminus of the tRNA^Gly^ gene (PYCH_t155). The size of GIs PYG3 and PYG4 was determined to be 13,628 and 12,135 bp, respectively. In these two regions, we did not find any tRNA genes or related integrase or transposase genes.

**Table 2:** Predicted GIs in the *P. yayanosii* genome.

GIs	Length (bp)	Island start	Island end	ORFs	tRNA	Integrase	Transposase	GC %
PYG1	21356	1321661	1343016	23	tRNA^Gln^	+	+	41.3
PYG2	17552	1238311	1255863	23	tRNA^Gly^	+	-	43.9
PYG3	13628	41713	55341	10	-	-	-	46.84
PYG4	12135	1566972	1579107	11	-	-	-	40.58
PYG5	9792	107966	117758	8	-	-	-	54.29
PYG6	7959	672922	680881	10	-	-	-	41.49
PYG7	7371	1590511	1597882	6	-	-	-	37.14
PYG8	6076	377786	383862	8	-	-	+	46.93
PYG9	5392	32347	37739	10	-	-	+	49.56
PYG10	5218	699830	705048	6	-	-	-	44.99
PYG11	4959	693429	698388	4	-	-	-	42.32
PYG12	4898	764428	769326	7	-	-	-	45.68
PYG13	4506	1114916	1119422	5	-	-	-	42.89
PYG14	4389	1065373	1069762	4	-	-	-	45.69
PYG15	4139	1093856	1097995	7	-	-	-	51.3

### PYG1 Is a Mosaic-Like Genomic Island with Assumed Multifunctional Roles

Functional annotations of 23 open reading frames (ORFs) in PYG1 were attempted through a combined analysis using BlastP and Pfam. However, three ORFs (PYCH_15130, PYCH_15200, and PYCH_15310) could not be matched with any protein in the databases (**Table 3**). According to the annotation and sequence alignment, the genes in PYG1 could tentatively be divided into five functional modules (**Figure 1A**).

**Table 3:** Predicted functions of the open reading frames (ORFs) in PYG1 and their homologs.

Module	G+C content (%)	ORF	Size (aa)	Proposed function of the BlastP best-hits	Source organism	*E*-Value	Identity%	Accession No.
I(1–3680)^a^	38.02	PYCH_15110	230	Site-specific recombinase	*Thermococcus barophilus* MP	4e-161	98	YP_004072041.1
		PYCH_15120	361	Hypothetical protein	*Thermococcus barophilus* MP	0	89	YP_004072042.1
		PYCH_15130	40	Hypothetical protein				
		PYCH_15140	103	Hypothetical protein	*Thermococcus barophilus* MP	2e-33	86	YP_004072043.1
		PYCH_15150	131	Hypothetical protein	*Thermococcus barophilus* MP	2e-67	82	YP_004072044.1
II(3681–9514)	40.26	PYCH_15160	468	McrBC 5-methylcytosine restriction system component	*Thermococcus gammatolerans* EJ3	2e-136	48	YP_002958818.1
		PYCH_15170^b^	491	GTPase subunit of restriction endonuclease	*Methanotorris formicicus*	3e-71	40	WP_007044636.1
		PYCH_15190	210	Methyladenine DNA glycosylase	*Clostridium ljungdahlii*	9e-37	38	YP_003781774.1
		PYCH_15200	81	Hypothetical protein				
		PYCH_15210	282	Hypothetical protein	*Thermococcus litoralis*	6e-49	54	WP_042692114.1
III(9515–15208)	43.99	PYCH_15220	689	Tetratricopeptide repeat domain-containing protein	*Thermococcus barophilus* MP (plasmid)	0	69	YP_004422849.1
		PYCH_15230	50	Hypothetical protein	*Thermococcus barophilus* MP (plasmid)	3e-04	69	YP_004422850.1
		PYCH_15240	311	ADP-ribosyl glycohydrolase	*Thermococcus barophilus* MP (plasmid)	0	92	YP_004422851.1
		PYCH_15250	113	γ-glutamylcyclotransferase	*Thermococcus barophilus* MP (plasmid)	3e-69	92	YP_004422852.1
		PYCH_15260	321	Class II glutamine amidotransferase	*Thermococcus barophilus* MP (plasmid)	0	87	YP_004422853.1
		PYCH_15270	404	Hypothetical protein	*Thermococcus barophilus* MP (plasmid)	0	90	YP_004422854.1
IV(15209–19321)	41.94	PYCH_15280	638	Hypothetical protein	*Hippea* sp. KM1	7e-125	37	WP_025209087.1
		PYCH_15290	296	ISA0963-5 transposase	*Archaeoglobus veneficus* SNP6	2e-78	47	YP_004341222.1
		PYCH_15300	88	Hypothetical protein	*Thermococcus eurythermalis*	1e-33	70	WP_050003915.1
		PYCH_15310	48	Hypothetical protein				
V(19322–21356)	39.85	PYCH_15320	208	Transcriptional regulator (Putative antitoxin AbiEi)	*Thermococcus barophilus* MP (Conserved domain DUF4095)	1e-127	93	YP_004072067.1
		PYCH_15330	253	Nucleotidyltransferase (Putative toxin AbiEii)	*Thermococcus barophilus* MP (Conserved domain COG2253)	1e-171	95	YP_004072068.1
		PYCH_15340	80	Hypothetical protein	*Thermococcus barophilus* MP	1e-39	93	YP_004072068.1
tRNA^Gln^		PYCH_t170						

*^a^ It contains PYG1-associated DR (1–43 bp) sequence (*attL*). ^b^ A GTPase subunit (PYCH_15170) is a pseudogene, because there is a base cytosine base redundancy at the 1,470-bp site of this gene that results in a frameshift mutation.*

The genes in module I include a putative integrase (PYCH_15110) and four other hypothetical proteins (PYCH15120 to PYCH15150), which showed a high similarity to the corresponding region of a predicted GI (named TBG1) in *T. barophilus* MP (Marteinsson et al., 1999).

In module II, there are two genes that were annotated as the putative McrBC 5-methylcytosine-dependent restriction endonuclease (PYCH_15160 and PYCH_15170) and a putative methyladenine DNA glycosylase (PYCH_15190). A potential GTPase subunit (PYCH_15170) might be a pseudogene because there is a redundant cytosine at the 1,470-bp site of this gene resulting in a frameshift mutation.

Module III is composed of six genes (PYCH_15220 to PYCH_15270), which showed high similarity to a corresponding region of a plasmid (pTBMP1) in *T. barophilus* MP. In this region, a tetratricopeptide repeat domain-containing protein (PYCH_15220) mediates protein-protein interactions or the assembly of multiprotein complexes (Zeytuni and Zarivach, 2012), an ADP-ribosyl glycohydrolase (PYCH_15240) catalyzes the chemical reaction that hydrolyses poly(ADP-ribose; Cuzzocrea and Wang, 2005), and a γ-glutamylcyclotransferase (PYCH_15250) and a class II glutamine amidotransferase (PYCH_15260) might participate in the γ-glutamyl cycle (Oakley et al., 2008).

In module IV (PYCH15280 to PYCH15310), PYCH_15290 and PYCH_15300 showed homology to a putative transposase and a type II restriction enzyme methylase subunit, respectively. The genes in module V (PYCH_15320 to tRNA^Gln^) encompass a pair of widespread prokaryotic orthologous group families, COG5340 (PYCH15320, domain of unknown function DUF4095) and COG2253 (PYCH15330, domain of unknown function DUF1814), which also showed high similarity to the corresponding region of a predicted GI (named TBG1) in *T. barophilus* MP and formed a toxin-antitoxin system (Dy et al., 2014).

### Putative DNA Mobilization Genes in PYG1

**FIGURE 2:**
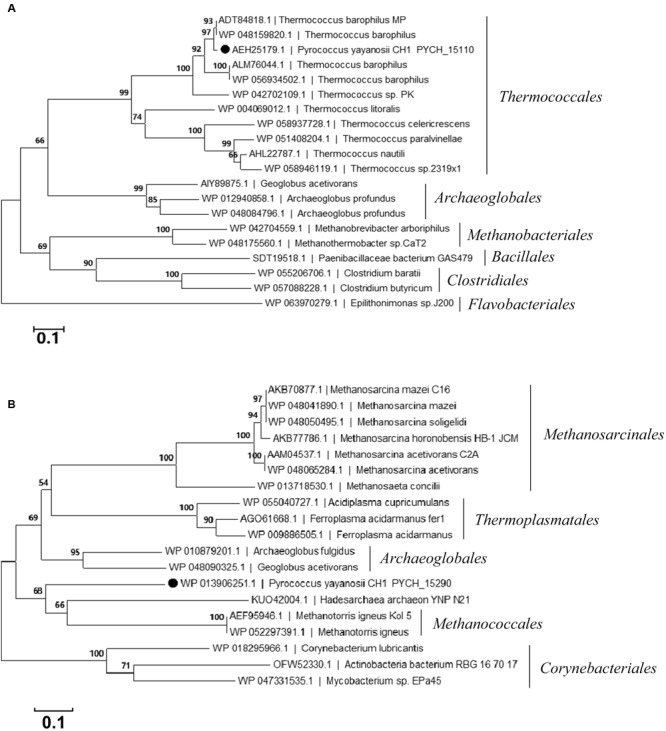
**Phylogenetic tree of the site-specific recombinase PYCH_15110 (A)** and transposase PYCH_15290 **(B)**. The filled circle represents the subject protein. The phylogenetic analysis was performed using the neighbor-joining algorithm. Bootstrap values (expressed as percentages of 1000 replications) are shown at branching points. The scale bar represents 0.1 substitutions per nucleotide position.

PYCH_15110 encodes a putative site-specific recombinase that belongs to the phage integrase family (tyrosine recombinase XerC/D). It contains a C-terminal catalytic domain of DNA breaking-rejoining enzymes. The phylogenetic analysis of PYCH_15110 revealed its evolutionary relationship with the site-specific recombinases derived from archaea. PYCH_15110 and its *Thermococcales* homologs (43–98% identity) formed a branch that was distantly separated from the other branches belonging to *Archaeoglobales, Methanobacteriales, Bacillales*, and *Clostridiales* (**Figure 2A**).

PYCH_15290 encodes a putative archaeal ISA0963-5-type transposase. It has three conserved domains, including an integrase core domain (*rve*) and a helix-turn-helix domain (HTH_23 and HTH_32) that is associated with DNA binding (Tran-Nguyen et al., 2008). The phylogenetic analysis of PYCH_15290 revealed its evolutionary relationship with the IS481 transposase family derived from archaea. PYCH_15290 shared close evolutionary relationships with archaeal transposases from *Methanococcales, Archaeoglobales, Thermoplasmatales*, and *Methanosarcinales* but not with any transposase in *Thermococcales* (**Figure 2B**).

### PYG1 Can Spontaneously Excise from the *P. yayanosii* A1 Chromosome

**FIGURE 3:**
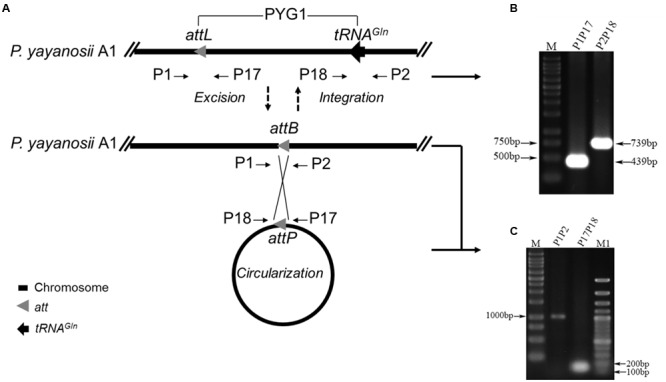
**PYG1 spontaneously excises from the *P. yayanosii* A1 chromosome. (A)** A schematic of the excision and circularization of PYG1. Thick straight lines represent the *P. yayanosii* A1 chromosome. The *attL* (a 43-bp DR is the left junction of PYG1), *attP* (following excision, both PYG1 ends close up to form the recombination site) and *attB* (the recombination site was re-formed on the chromosome after excision of PYG1) sites are shown as gray triangles, tRNA^Gln^ is shown as black arrowheads, and the primer annealing sites are represented by short arrows. **(B)** Two PCR products (439 and 739 bp) were amplified by the primer pairs P1, P17 and P2, P18, respectively. The results showed that PYG1 exists in the chromosome. **(C)** PCR analysis of *P. yayanosii* A1 genomic DNA using primers P1 and P2 detected a 1,018-bp fragment, and primers P17 and P18 detected a 155-bp fragment, suggesting the existence of excised and circularized forms of PYG1. All of these PCR products were confirmed by DNA sequencing.

Diagnostic primer sets targeting the *attL* sites (P1 and P17) and tRNA^Gln^ gene (P18 and P2) were used to detect whether PYG1 could excise from the chromosome (**Figures 3A,B**). The PCR amplification of template chromosome DNA using primers P1 and P2 generated a 1,018-bp DNA fragment, which became detectable after PYG1 was excised from the chromosome (**Figure 3C**). The other primer set, P17 and P18, yielded a 155-bp PCR amplification product (**Figure 3C** and Supplementary Figure S3), suggesting that the excised PYG1 formed an episomal ring (**Figure 3C**). These results were confirmed by DNA sequencing and indicated that the integrated GI PYG1 spontaneously excised from the chromosome of *P. yayanosii* A1. The ratio of the cells containing the circular form of excised PYG1 was evaluated using real-time PCR. The copy number of the circular PYG1 (determined through PCR using primers P17 and P18) was compared with the copy number of the reference gene 16S rRNA (determined through PCR using primers q16S F/R). The observed relative ratio of cells harboring the spontaneously excised circular form of PYG1 was 1.2 × 10^-8^ (Supplementary Figure S4).

### Role of the Integrase and *att* Site in the Process of Site-Specific Excision and Integration of PYG1

**FIGURE 4:**
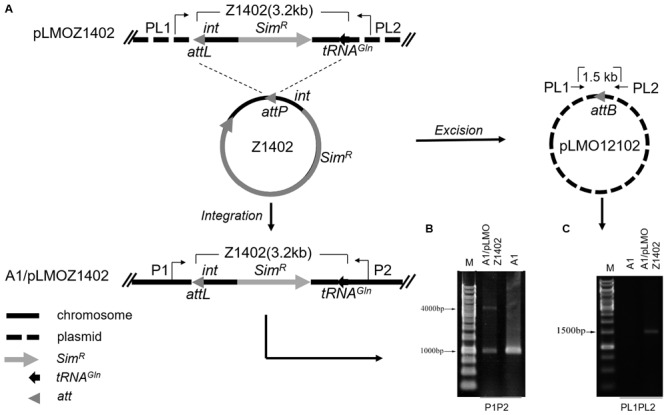
**The int gene could mediate the integration and excision of mini-islands in *P. yayanosii* A1 at the att site. (A)** A schematic of the integration and circularization of mini-island Z1402 in *P. yayanosii* A1. The mini-island Z1402 (thick solid-line circle) excised from plasmid pLMOZ1402 and integrated into *P. yayanosii* A1 at the attL site (*attL, attP*, and *attB* are shown as gray triangles), resulting a circular plasmid pLMO12102 (thick dashed line circle). **(B)** PCR analysis of the genomic DNA of transformants (A1/pLMOZ1402) using primers P1 and P2 detected a 4,127-bp fragment, confirming the integration of the mini-island Z1402 in *P. yayanosii* A1. The 1,018-bp fragment indicated that the integrative PYG1 and Z1402 can excise from the chromosome. **(C)** Primers PL1 and PL2 were used to determine whether the recombination site (*attB*) was formed on the plasmid after excision of Z1402. A 1,550-bp PCR product was observed, suggesting that the mini-island Z1402 could be excised from plasmid pLMOZ1402. *P. yayanosii* A1 was used as a control sample in these experiments.

To confirm the function of the putative integrase gene (*int*, PYCH_15110) and the requirement of the *att* site in the excision and integration of PYG1, a series of mini-islands were constructed (**Table 1**). The mini-island Z1402, harboring the *att* site, the *int* gene, tRNA^Gln^, and other PYG1-internal genes was replaced with the *Sim*^R^ cassette (**Figure 4A**). The recombinant plasmid pLMOZ1402 was constructed after the insertion of Z1402 into the *E. coli–P. yayanosii* shuttle vector pLMO12102 carrying a pUC18 replicon, an ampicillin resistance gene and a pGT5 plasmid replication region. The plasmid pLMOZ1402 was introduced into *P. yayanosii* A1. The total DNA from the simvastatin-resistant transformant was extracted and used as a template in the PCR amplification. A 4,127-bp PCR product obtained using primers P1 and P2 indicated that the mini-island Z1402 could specifically integrate into the chromosome of *P. yayanosii* A1 at the *att* site (**Figure 4B**). We also obtained a 1,550-bp PCR product (the sequence of the plasmid) using primers PL1 and PL2, showing that the mini-island Z1402 could excise from plasmid pLMOZ1402 at the *attB* site (**Figure 4C**). In addition, the 1,018-bp fragment was amplified again both in *P. yayanosii* A1 and transformant A1/pLMOZ1402. This result indicated that the integrative PYG1 and Z1402 could excise from the chromosome (**Figure 4B**).

Whether pLMOZ1404 (lacking the *att* sequence) could be integrated into the *P. yayanosii* A1 genome was examined using the same primers, P1 and P2 (Supplementary Figure S5A). We did not obtain an expected 4,084-bp PCR product (the 43-bp *att* site was reduced from the integrative Z1402); only the 1,018-bp product was amplified (PYG1 excised from the chromosome), indicating that the mini-island Z1404 could not integrate into the genome of *P. yayanosii* A1 (Supplementary Figure S5B). Primers PL1 and PL2 also only amplified a 4,298-bp product (the sequence of the plasmid was included in the amplified product, so the length of the product is longer than Z1404; Supplementary Figure S5D), demonstrating that the mini-island Z1404 could not be excised from plasmid pLMOZ1404.

Plasmid pLMOZ1405, carrying the mini-island Z1405 (lacking the putative *int* gene PYCH_15110), was transformed into the mutant strain Δ*int* (Supplementary Figure S5A). Only a 3,753-bp PCR product (lacking the putative *int* gene) was obtained using primers PL1 and PL2, indicating that the mini-island Z1405 is not excised from plasmid pLMOZ1405 (Supplementary Figure S5E). However, the 1,018-bp PCR product was still detected in the Δ*int* and Δ*int*/pLMOZ1405 strains (Supplementary Figure S5C). This result showed that the mini-island Z1405 cannot integrate into the genome of Δ*int*. Based on the results above, the integrase *int* and the *att* site are required for the excision and integration of the GI PYG1.

### Removal of PYG1 Affected the Growth of *P. yayanosii*

**FIGURE 5:**
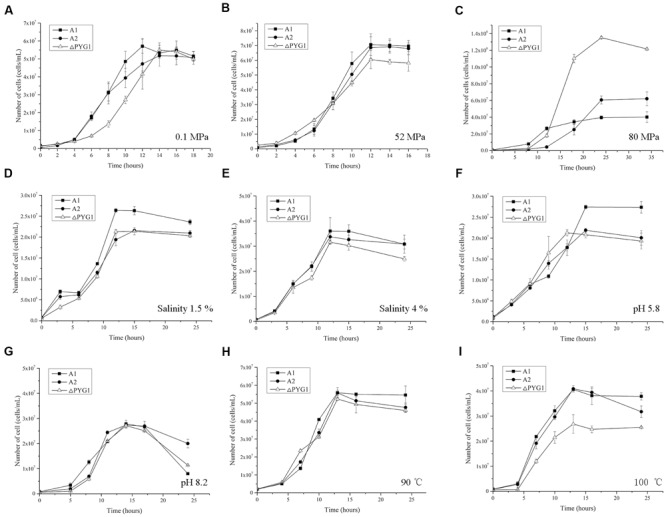
**Growth characteristics of the ΔPYG1, A1, and A2 strains cultivated in modified TRM under different conditions.** The cell numbers were counted after 12 h cultivation. Each value is the average of three measurements, and the error bars indicate the SD. **(A)** 0.1 MPa, 95°C, salinity 3%, pH 7; **(B)** 52 MPa, 95°C, salinity 3%, pH 7; **(C)** 80 MPa, 95°C, salinity 3%, pH 7; **(D)** 0.1 MPa, 95°C, salinity 1.5%, pH 7; **(E)** 0.1 MPa, 95°C, salinity 4%, pH 7; **(F)** 0.1 MPa, 95°C, salinity 3%, pH 5.8; **(G)** 0.1 MPa, 95°C, salinity 3%, pH 8.2; **(H)** 0.1 MPa, 90°C, salinity 3%, pH 7; **(I)** 0.1 MPa, 100°C, salinity 3%, pH 7.

To assess the physiological importance of PYG1, the growth curve of the PYG1 knockout strain (*Δ*PYG1) was tested in TRM under two cultivation conditions. In the condition of 0.1 MPa, 95°C, and salinity 3% (**Figure 5A**), the *Δ*PYG1 mutant strain showed a significant delay in the logarithmic growth phase, but the biomass of *Δ*PYG1 and the parental strain A2 and A1 were similar in the stationary growth phase. In the condition of 52 MPa, 98°C, and salinity 3% (**Figure 5B**), the growth curve of *Δ*PYG1 was similar to the control groups. Interestingly, the biomass of the mutant strain increased significantly compared to *P. yayanosii* A1 and A2 under the condition of 80 MPa, 95°C, and salinity 3%. In addition, the time of the logarithmic growth of these three strains lagged behind the optimal conditions (**Figure 5C**), while the specific growth rate of ΔPYG1 is higher than *P. yayanosii* A1 and A2 under 80 MPa (Supplementary Table S2).

Meanwhile, the growth of *Δ*PYG1 was challenged under stress conditions by modifying the salinity and pH of the TRM or increasing the cultivation temperature. No obvious growth differences were observed in the medium that had extreme salinity (**Figures 5D,E**), pH (**Figures 5F,G**) or low temperature (**Figure 5H**). However, the growth curve of *Δ*PYG1, A2, and A1 showed significant differences under higher temperature stress. Under 100°C conditions, impaired growth of the mutant strain ΔPYG1 was observed in both the logarithmic and stationary growth phases (**Figure 5I**).

### Transcriptional Analysis of the Module III Genes

We analyzed the transcription levels of the genes in module III in *P. yayanosii* A1 under different temperatures (90, 95, and 100°C) and different pressures (0.1 MPa, 52 MP, and 70 MPa) by using relative real-time PCR. The transcription levels of every gene under optimal temperature (95°C) and optimal pressure (52 MPa) was used as a reference.

**FIGURE 6:**
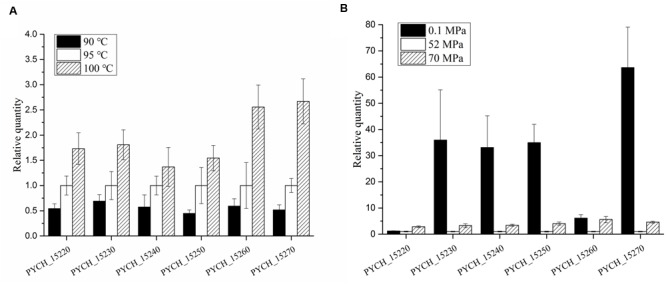
**Transcription levels of genes in module III of PYG1 under different temperatures and pressures.** The transcription level under the condition of 95°C and 52 MPa were assigned as references. **(A)** The transcription levels of the six genes were up-regulated under high temperature conditions. PYCH_15260 and PYCH_15270 under 100°C showed obvious differences compared with 95°C. **(B)** Under high pressure stress (70 MPa), the transcription levels of every gene were up-regulated compared with the optimal pressure (52 MPa). The transcription levels of PYCH_15230, PYCH_15240, PYCH_15250, and PYCH_15270 were significantly up-regulated at 0.1 MPa compared to 52 and 70 MPa.

All six of the genes in module III were transcriptionally up-regulated under higher temperature (**Figure 6A**). The fold changes in transcription were more pronounced under high temperature stress (100°C) than under low temperature stress (90°C). Under high pressure stress (70 MPa), the transcription levels of every gene were up-regulated compared with the optimal pressure (52 MPa). More interestingly, the transcription levels of PYCH_15230, PYCH_15240, PYCH_15250, and PYCH_15270 were significantly up-regulated at 0.1 MPa compared to 52 MPa and 70 MPa (**Figure 6B**).

## Discussion

In this study, we characterized the largest GI PYG1 in *P. yayanosii*, which showed high sequence similarity to its counterparts within *T. barophilus* MP as either a GI TBG1 in the chromosome or a DNA fragment in plasmid pTBMP1 (Marteinsson et al., 1999; Vannier et al., 2011). Moreover, both ends of the GIs PYG1 and TBG1 were aligned as functional modules, reflecting a GI frame that is capable of site-specific integration.

We found gene arrangements similar to module III of PYG1 in a number of archaea, including *P. abyssi* GE5 (PAB1037 and PAB1036) and *P. furiosus* DSM 3638 (PF1316 and PF1317), *Methanotorris igneus* Kol 5 (Metig1401 and Metig1402), *Methanocaldococcus* sp. FS406-22 (MFS0524 and MFS0525), and *Methanocaldococcus jannaschii* DSM 2661 (MJ1514 and MJ1515; Supplementary Figure S2). Almost all of the genes in module III have overlapping base pairs with a flanking coding sequence. There are four-base pair overlaps between PYCH_15210 and PYCH15220, PYCH_15230 and PYCH_15240, and PYCH_15250 and PYCH_15260. PYCH_15240 and PYCH_15250 are separated by only two intergenic base pairs. Such a tightly organized gene cluster suggests that module III might exert its effect coordinately.

The horizontal transfer of GIs is often initiated through the excision of a linear form from the chromosome to produce a circular, mobilizable episome (Dobrindt et al., 2004). PYG1 can spontaneously excise from the chromosome, and the cyclization rate of PYG1 was maintained at a lower level (1.2 × 10^-8^). These results suggested the functional importance of maintaining PYG1 and indicated that there may be a maintenance mechanism. Defense genes contribute to the maintenance of mobile genetic elements in bacterial or archaeal populations (Wozniak and Waldor, 2009; Vasu and Nagaraja, 2013; Dy et al., 2014). Here, we assumed that the putative restriction-modification system (PYCH_15160-PYCH_15170) and toxin-antitoxin system (PYCH_15320-PYCH_15330) encoded by PYG1 might have a role in maintaining cyclization at a low frequency.

The direct relevance of the *att* site and *int* gene for mediating the site-specific excision and integration of PYG1 into the *P. yayanosii* A1 chromosome was confirmed using a series of mini-islands constructed with *int* and *Sim*^R^ cassettes sandwiched between *attL* and tRNA^Gln^ (PYCH_t170) in a shuttle plasmid. However, the PYG1 was still excised from the chromosome of the *int* gene (PYCH_15110) disruption mutant. The *int* gene was predicted to belong to a XerC/D recombinase family. In the *P. yayanosii* genome, another gene (PYCH_00910, GenBank accession No. WP_013904862.1) was annotated as a putative XerC-like integrase. We found that the XerC (PYCH_00910) integrase was obviously up-regulated in the mutant strain Δ*int* (Data not shown). In *Neisseria gonorrhoeae*, XerC/D proteins mediated the excision of the gonococcal genetic island (GGI) from the genome (Midonet and Barre, 2016). We speculated that PYCH_15110 and PYCH_00910 are functionally complementary, and they might be associated with the integration and excision of PYG1 in *P. yayanosii*.

The removal of PYG1 provided an opportunity to determine the physiological function of this GI in *P. yayanosii.* Not surprisingly, PYG1 was proven to be a dispensable genetic element in most environmental conditions. GIs could confer an adaptive advantage in some stress conditions. GI genes can respond to environmental signals, such as pH, osmolality, temperature, cell density, or the concentration of specific elements (Deiwick et al., 1999; Banos et al., 2009). We found that high temperature (100°C) significantly inhibited the growth of the mutant strain ΔPYG1. In contrast, the mutant strain ΔPYG1 grew better than A1 and A2 under high pressure (80 MPa). Whether the upper cardinal temperature for growth could be extended or not in the mutant strain ΔPYG1 under high pressure (80 MPa) is an interesting point.

Temperature is supposed to be the core environmental parameter that selects microbial adaptation process (Xiao and Zhang, 2014). High pressure and low temperature share similar effects on protein synthesis and membrane structure (Bartlett, 2002). Pressure is known to increase the upper temperature for growth of many bacteria isolated from the cold deep-sea (Yayanos, 1986), as well as accelerate the growth rate of thermophilic methanogen (Miller et al., 1988). Isolated as the first obligate piezo-hyperthermophilic archaeon, *P. yayanosii* demonstrated optimal growth under 52 MPa, which is higher than the hydrostatic pressure equivalent to its habitat at a depth of 4,100 m. We assumed that PYG1 conferred the adaption to higher temperature with a compensation of reduced pressure tolerance in *P. yayanosii*. The physiological tradeoff of high temperature and high pressure, which became more pronounced after removal of PYG1, should be examined more carefully.

Each of the genes in module III of PYG1 showed high similarity to its counterpart that resided on a plasmid (pTBMP1) in *T. barophilus* MP. We assume that the HGT of module III into either *T. barophilus* or *P. yayanosii* could benefit these two piezophilic hyperthermophiles. The transcription levels of the genes in module III (PYCH_15220 to PYCH_15270) were up-regulated under low pressure (0.1 MPa, 95°C) and high pressure (70 MPa, 95°C) conditions compared with the optimal conditions (52 MPa, 95°C). These results were consistent with the transcriptomic study of *P. yayanosii*, in which it was shown that the transcription levels of PYCH_15210, PYCH_15270, and PYCH_15290 in PYG1 under 20 and 80 MPa conditions were higher than those observed under 52 MPa conditions (Michoud and Jebbar, 2016). Under high temperature stress, the expression of PYCH_15260 and PYCH_15270 showed obvious up-regulation at 100°C. Interestingly, these two genes shared a high identity with PF1317 and PF1316 of *P. furiosus*, respectively (Supplementary Figure S2). PF1316 and PF1317 were reported to be members of a large gene cluster that was significantly up-regulated in response to peroxide stress (Strand et al., 2010), but the functional annotations of PF1316 and PF1317 are still unclear.

The tuning of overall gene expression, the expression of HHP stress-specific genes and the adaptation of the biomolecular structure are three main mechanisms to explain the ability of piezophiles to grow best under HHP (Oger and Jebbar, 2010). High pressure influences on gene and protein expression (Bartlett et al., 1995). Increasing of pressure enhanced activity and stability of a hyperthermophilic protease (Michels and Clark, 1997). Metabolic adjustment at the global scale has been shown to be a response to pressure stress in *T. barophilus* (Vannier et al., 2015). Moreover, HHP increases amino acid requirements in *T. barophilus* MP (Cario et al., 2015). Several amino acid biosynthesis pathways are missing in the *P. yayanosii* genome (Michoud and Jebbar, 2016). PYCH_15260 is annotated as a class II glutamine amidotransferase. This enzyme is believed to be involved in the biosynthesis of glucosamine, nucleotides, and amino acids (tryptophan, histidine, asparagine, and glutamate), among other molecules (Massiere and Badet-Denisot, 1998).

The present study provides the first insights into the physiological function of the largest GI PYG1 in *P. yayanosii*, which affects the host’s high temperature and HHP adaptation. Moreover, characterizing the excision and integration of PYG1 mediated by *att* and the integrase could lead to the development of novel site-specific integrative genetic tools for this group of piezophilic hyperthermophilic archaea.

## Author Contributions

XX and JX designed the experiments; ZL and XL performed the experiments; and ZL and JX drafted the manuscript. All authors discussed and reviewed the manuscript.

## Conflict of Interest Statement

The authors declare that the research was conducted in the absence of any commercial or financial relationships that could be construed as a potential conflict of interest.

## References

[B1] Banos R. C. Vivero A. Aznar S. Garcia J. Pons M. Madrid C. (2009). Differential regulation of horizontally acquired and core genome genes by the bacterial modulator H-NS. *PLoS Genet.* 5:e1000513 10.1371/journal.pgen.1000513 PMC2686267 19521501

[B2] Bartlett D. H. (2002). Pressure effects on in vivo microbial processes. *Biochim. Biophys. Acta* 1595 367–381. 10.1016/S0167-4838(01)00357-0 11983409

[B3] Bartlett D. H. Kato C. Horikoshi K. (1995). High pressure influences on gene and protein expression. *Res. Microbiol.* 146 697–706. 10.1016/0923-2508(96)81066-7 8584792

[B4] Bertoldo C. Antranikian G. (2006). “The order thermococcales,” in *The Prokaryotes* 3rd Edn Vol. 3 eds Dworkin M. Falkow S. Rosenberg E. Schleifer K.-H. Stackebrandt E. (New York, NY: Springer) 69–81. 10.1007/0-387-30743-5_5

[B5] Birrien J. L. Zeng X. Jebbar M. Cambon-Bonavita M. A. Querellou J. Oger P. (2011). *Pyrococcus yayanosii* sp. nov., an obligate piezophilic hyperthermophilic archaeon isolated from a deep-sea hydrothermal vent. *Int. J. Syst. Evol. Microbiol.* 61 2827–2831. 10.1099/ijs.0.024653-0 21239564

[B6] Campanaro S. Vezzi A. Vitulo N. Lauro F. M. D’angelo M. Simonato F. (2005). Laterally transferred elements and high pressure adaptation in *Photobacterium profundum* strains. *BMC Genomics* 6:122 10.1186/1471-2164-6-122 PMC1239915 16162277

[B7] Cario A. Lormieres F. Xiang X. Oger P. (2015). High hydrostatic pressure increases amino acid requirements in the piezo-hyperthermophilic archaeon *Thermococcus barophilus*. *Res. Microbiol.* 166 710–716. 10.1016/j.resmic.2015.07.004 26226334

[B8] Cuzzocrea S. Wang Z. Q. (2005). Role of poly(ADP-ribose) glycohydrolase (PARG) in shock, ischemia and reperfusion. *Pharmacol. Res.* 52 100–108. 10.1016/j.phrs.2005.02.009 15911338

[B9] Darmon E. Leach D. R. (2014). Bacterial genome instability. *Microbiol. Mol. Biol. Rev.* 78 1–39. 10.1128/MMBR.00035-13 24600039 PMC3957733

[B10] Deiwick J. Nikolaus T. Erdogan S. Hensel M. (1999). Environmental regulation of *Salmonella* pathogenicity island 2 gene expression. *Mol. Microbiol.* 31 1759–1773. 10.1046/j.1365-2958.1999.01312.x 10209748

[B11] Dobrindt U. Hochhut B. Hentschel U. Hacker J. (2004). Genomic islands in pathogenic and environmental microorganisms. *Nat. Rev. Microbiol.* 2 414–424. 10.1038/nrmicro884 15100694

[B12] Dy R. L. Przybilski R. Semeijn K. Salmond G. P. Fineran P. C. (2014). A widespread bacteriophage abortive infection system functions through a Type IV toxin-antitoxin mechanism. *Nucleic Acids Res.* 42 4590–4605. 10.1093/nar/gkt1419 24465005 PMC3985639

[B13] Ferrera I. Reysenbach A.-L. (2007). *Thermophiles. Encyclopedia of Life Sciences.* Hoboken, NJ: John Wiley & Sons, Ltd 10.1002/9780470015902.a0000406

[B14] Fukui T. Atomi H. Kanai T. Matsumi R. Fujiwara S. Imanaka T. (2005). Complete genome sequence of the hyperthermophilic archaeon *Thermococcus kodakaraensis* KOD1 and comparison with *Pyrococcus* genomes. *Genome Res.* 15 352–363. 10.1101/gr.3003105 15710748 PMC551561

[B15] He X. Ou H. Y. Yu Q. Zhou X. Wu J. Liang J. (2007). Analysis of a genomic island housing genes for DNA S-modification system in Streptomyces lividans 66 and its counterparts in other distantly related bacteria. *Mol. Microbiol.* 65 1034–1048. 10.1111/j.1365-2958.2007.05846.x 17640271

[B16] Jebbar M. Franzetti B. Girard E. Oger P. (2015). Microbial diversity and adaptation to high hydrostatic pressure in deep-sea hydrothermal vents prokaryotes. *Extremophiles* 19 721–740. 10.1007/s00792-015-0760-3 26101015

[B17] Juhas M. Van Der Meer J. R. Gaillard M. Harding R. M. Hood D. W. Crook D. W. (2009). Genomic islands: tools of bacterial horizontal gene transfer and evolution. *FEMS Microbiol. Rev.* 33 376–393. 10.1111/j.1574-6976.2008.00136.x 19178566 PMC2704930

[B18] Jun X. Lupeng L. Minjuan X. Oger P. Fengping W. Jebbar M. (2011). Complete genome sequence of the obligate piezophilic hyperthermophilic archaeon *Pyrococcus yayanosii* CH1. *J. Bacteriol.* 193 4297–4298. 10.1128/JB.05345-11 21705594 PMC3147706

[B19] Langille M. G. I. Brinkman F. S. L. (2009). IslandViewer: an integrated interface for computational identification and visualization of genomic islands. *Bioinformatics* 25 664–665. 10.1093/bioinformatics/btp030 19151094 PMC2647836

[B20] Li X. Fu L. Li Z. Ma X. Xiao X. Xu J. (2015). Genetic tools for the piezophilic hyperthermophilic archaeon *Pyrococcus yayanosii*. *Extremophiles* 19 59–67. 10.1007/s00792-014-0705-2 25391810

[B21] Makarova K. S. Wolf Y. I. Snir S. Koonin E. V. (2011). Defense islands in bacterial and archaeal genomes and prediction of novel defense systems. *J. Bacteriol.* 193 6039–6056. 10.1128/JB.05535-11 21908672 PMC3194920

[B22] Marteinsson V. T. Birrien J. L. Reysenbach A. L. Vernet M. Marie D. Gambacorta A. (1999). *Thermococcus barophilus* sp. nov., a new barophilic and hyperthermophilic archaeon isolated under high hydrostatic pressure from a deep-sea hydrothermal vent. *Int. J. Syst. Bacteriol.* 49(Pt 2) 351–359. 10.1099/00207713-49-2-351 10319455

[B23] Martin-Cuadrado A. B. Pasic L. Rodriguez-Valera F. (2015). Diversity of the cell-wall associated genomic island of the archaeon *Haloquadratum walsbyi*. *BMC Genomics* 16:603 10.1186/s12864-015-1794-8 PMC4535781 26268990

[B24] Massiere F. Badet-Denisot M. A. (1998). The mechanism of glutamine-dependent amidotransferases. *Cell. Mol. Life Sci.* 54 205–222. 10.1007/s000180050145 9575335 PMC11147313

[B25] Michels P. C. Clark D. S. (1997). Pressure-enhanced activity and stability of a hyperthermophilic protease from a deep-sea methanogen. *Appl. Environ. Microbiol.* 63 3985–3991. 16535711 10.1128/aem.63.10.3985-3991.1997 PMC1389267

[B26] Michoud G. Jebbar M. (2016). High hydrostatic pressure adaptive strategies in an obligate piezophile *Pyrococcus yayanosii*. *Sci. Rep.* 6:27289 10.1038/srep27289 PMC4890121 27250364

[B27] Midonet C. Barre F. X. (2016). How Xer-exploiting mobile elements overcome cellular control. *Proc. Natl. Acad. Sci. U.S.A.* 113 8343–8345. 10.1073/pnas.1608539113 27422553 PMC4968745

[B28] Miller J. F. Shah N. N. Nelson C. M. Ludlow J. M. Clark D. S. (1988). Pressure and temperature effects on growth and methane production of the extreme thermophile *Methanococcus jannaschii*. *Appl. Environ. Microbiol.* 54 3039–3042. 16347794 10.1128/aem.54.12.3039-3042.1988 PMC204424

[B29] Oakley A. J. Yamada T. Liu D. Coggan M. Clark A. G. Board P. G. (2008). The identification and structural characterization of C7orf24 as γ-glutamyl cyclotransferase: an essential enzyme in the γ-glutamyl cycle. *J. Biol. Chem.* 283 22031–22042. 10.1074/jbc.M803623200 18515354

[B30] Oger P. M. Jebbar M. (2010). The many ways of coping with pressure. *Res. Microbiol.* 161 799–809. 10.1016/j.resmic.2010.09.017 21035541

[B31] Polz M. F. Alm E. J. Hanage W. P. (2013). Horizontal gene transfer and the evolution of bacterial and archaeal population structure. *Trends Genet.* 29 170–175. 10.1016/j.tig.2012.12.006 23332119 PMC3760709

[B32] Reysenbach A. L. Liu Y. Banta A. B. Beveridge T. J. Kirshtein J. D. Schouten S. (2006). A ubiquitous thermoacidophilic archaeon from deep-sea hydrothermal vents. *Nature* 442 444–447. 10.1038/nature04921 16871216

[B33] Sobecky P. A. Hazen T. H. (2009). Horizontal gene transfer and mobile genetic elements in marine systems. *Methods Mol. Biol.* 532 435–453. 10.1007/978-1-60327-853-9_25 19271200

[B34] Stetter K. O. (2006). History of discovery of the first hyperthermophiles. *Extremophiles* 10 357–362. 10.1007/s00792-006-0012-7 16941067

[B35] Strand K. R. Sun C. Li T. Jenney F. E. Jr. Schut G. J. Adams M. W. (2010). Oxidative stress protection and the repair response to hydrogen peroxide in the hyperthermophilic archaeon *Pyrococcus furiosus* and in related species. *Arch. Microbiol.* 192 447–459. 10.1007/s00203-010-0570-z 20379702

[B36] Tagashira K. Fukuda W. Matsubara M. Kanai T. Atomi H. Imanaka T. (2013). Genetic studies on the virus-like regions in the genome of hyperthermophilic archaeon, *Thermococcus kodakarensis. Extremophiles* 17 153–160. 10.1007/s00792-012-0504-6 23224520

[B37] Tran-Nguyen L. T. Kube M. Schneider B. Reinhardt R. Gibb K. S. (2008). Comparative genome analysis of “*Candidatus Phytoplasma australiense*” (subgroup tuf-Australia I; rp-A) and “*Ca. Phytoplasma asteris*” strains OY-M and AY-WB. *J. Bacteriol.* 190 3979–3991. 10.1128/JB.01301-07 18359806 PMC2395047

[B38] van Wolferen M. Ajon M. Driessen A. J. Albers S. V. (2013). How hyperthermophiles adapt to change their lives: DNA exchange in extreme conditions. *Extremophiles* 17 545–563. 10.1007/s00792-013-0552-6 23712907

[B39] Vannier P. Marteinsson V. T. Fridjonsson O. H. Oger P. Jebbar M. (2011). Complete genome sequence of the hyperthermophilic, piezophilic, heterotrophic, and carboxydotrophic archaeon *Thermococcus barophilus* MP. *J. Bacteriol.* 193 1481–1482. 10.1128/JB.01490-10 21217005 PMC3067617

[B40] Vannier P. Michoud G. Oger P. Marteinsson V. Jebbar M. (2015). Genome expression of *Thermococcus barophilus* and *Thermococcus kodakarensis* in response to different hydrostatic pressure conditions. *Res. Microbiol.* 166 717–725. 10.1016/j.resmic.2015.07.006 26239966

[B41] Vasu K. Nagaraja V. (2013). Diverse functions of restriction-modification systems in addition to cellular defense. *Microbiol. Mol. Biol. Rev.* 77 53–72. 10.1128/MMBR.00044-12 23471617 PMC3591985

[B42] White J. R. Escobar-Paramo P. Mongodin E. F. Nelson K. E. Diruggiero J. (2008). Extensive genome rearrangements and multiple horizontal gene transfers in a population of *Pyrococcus* isolates from Vulcano Island, Italy. *Appl. Environ. Microbiol.* 74 6447–6451. 10.1128/AEM.01024-08 18723649 PMC2570278

[B43] Wozniak R. A. Waldor M. K. (2009). A toxin-antitoxin system promotes the maintenance of an integrative conjugative element. *PLoS Genet.* 5:e1000439 10.1371/journal.pgen.1000439 PMC2654960 19325886

[B44] Xiao X. Zhang Y. (2014). Life in extreme environments: approaches to study life-environment co-evolutionary strategies. *Sci. China Earth Sci.* 57 869–877. 10.1007/s11430-014-4858-8

[B45] Yayanos A. A. (1986). Evolutional and ecological implications of the properties of deep-sea barophilic bacteria. *Proc. Natl. Acad. Sci. U.S.A.* 83 9542–9546. 10.1073/pnas.83.24.9542 16593790 PMC387176

[B46] Zeng X. Birrien J. L. Fouquet Y. Cherkashov G. Jebbar M. Querellou J. (2009). *Pyrococcus* CH1, an obligate piezophilic hyperthermophile: extending the upper pressure-temperature limits for life. *ISME J.* 3 873–876. 10.1038/ismej.2009.21 19295639

[B47] Zeytuni N. Zarivach R. (2012). Structural and functional discussion of the tetra-trico-peptide repeat, a protein interaction module. *Structure* 20 397–405. 10.1016/j.str.2012.01.006. 22404999

