# Supplementary material for: An Integrative Genomic Island Affects the Adaptations of the Piezophilic Hyperthermophilic Archaeon Pyrococcus yayanosii to High Temperature and High Hydrostatic Pressure
Source: Front Microbiol. 2016 Nov 29;7:1927. doi: 10.3389/fmicb.2016.01927 (PMC5126054; doi:10.3389/fmicb.2016.01927)
Supplement: Supplementary file 1 [file Data_Sheet_1.DOCX]

Supplementary Material

**An Integrative Genomic Island Affects the Adaptations of Piezophilic Hyperthermophilic Archaeon *Pyrococcus yayanosii* to High Temperature and High Hydrostatic Pressure**

**Zhen Li, Xuegong Li, Xiang Xiao and Jun Xu***

***Correspondence:** Jun Xu: xujunn@sjtu.edu.cn

# 1 Supplementary Figures and Tables

## 1.1 Supplementary Figures

**
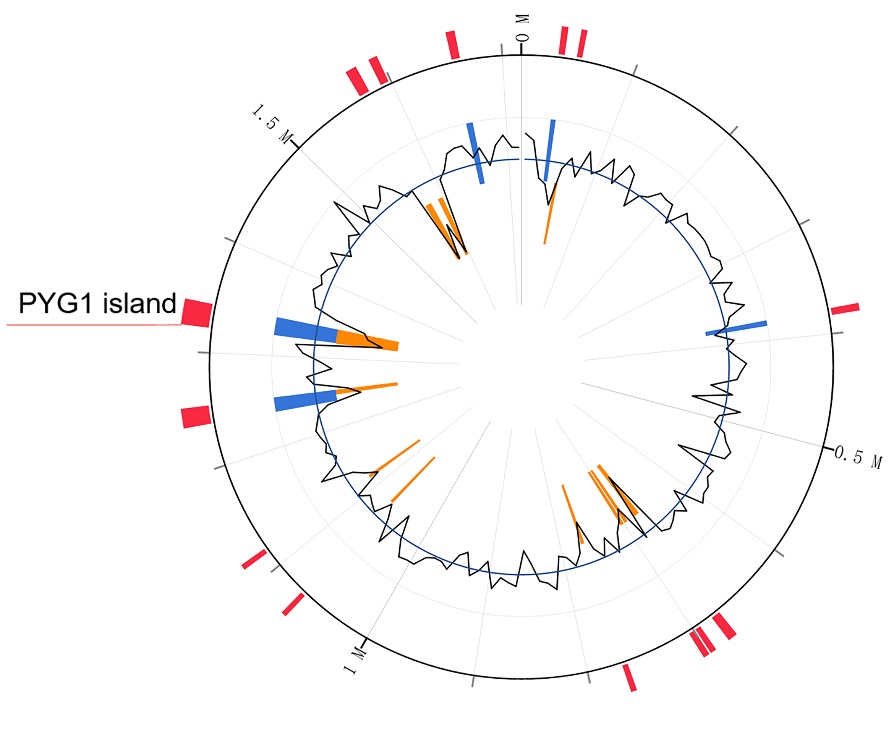
**

**Supplementary Figure 1.** IslandViewer analyses of the *P. yayanosii* chromosome, with colored regions indicating GIs. Red, blue and orange, respectively, represent the results predicted by the Integrated, SIGI-HMM and IslandPath-DIMOB prediction methods. The genomic island PYG1 was marked with a red line.


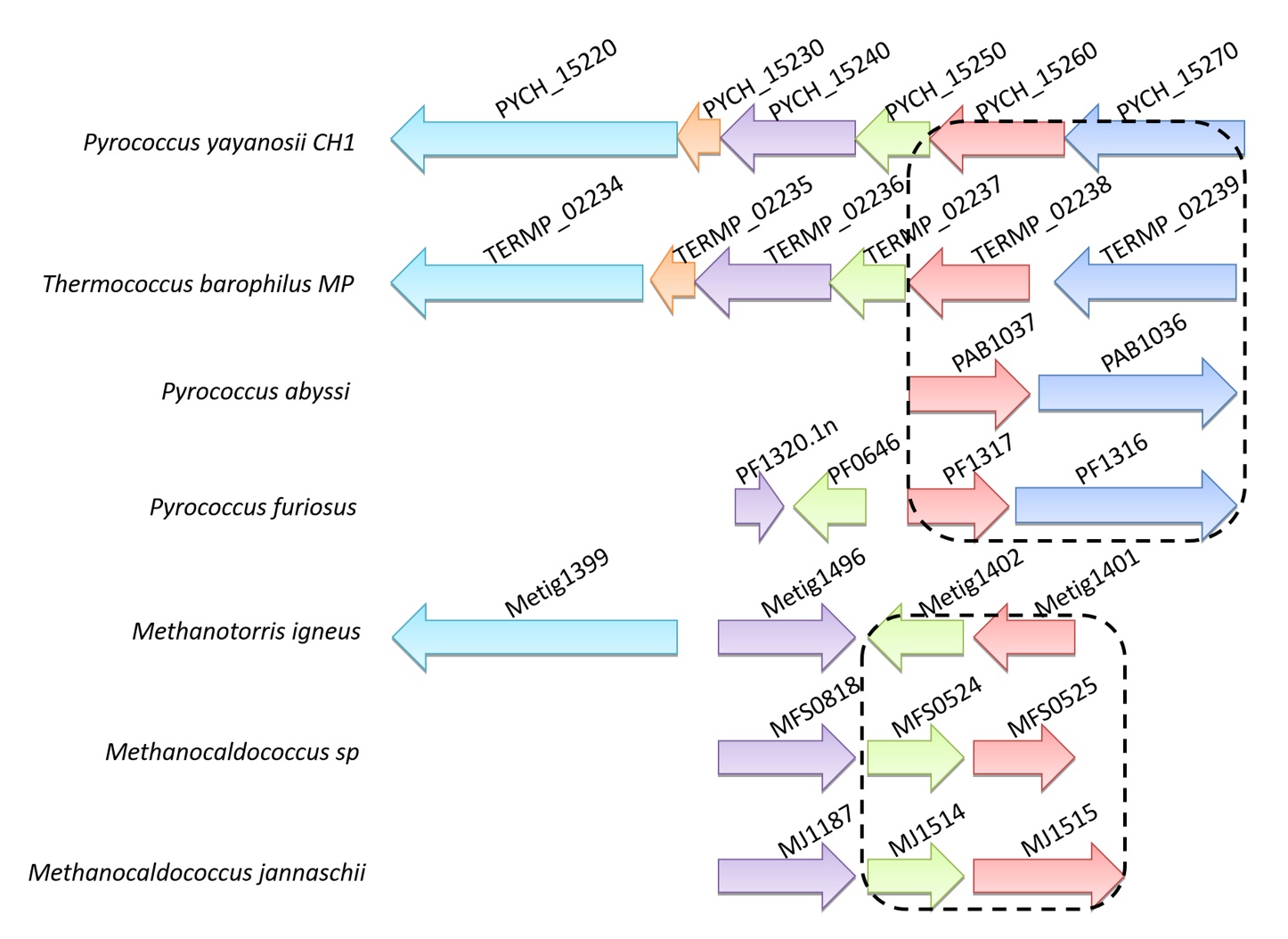


**Supplementary Figure 2.** Maps of a putative functional gene cluster consisting of six genes in PYG1 that shares high identity with a plasmid of *T. barophilus* MP. Homologous genes from *T. barophilus* MP are located on plasmid pTBMP1. All of the other genes are from the chromosomes of *P. abyssi* GE5 and *P. furiosus* DSM 3638 and *Methanotorris igneus* Kol 5, *Methanocaldococcus sp.* FS406-22 and *Methanocaldococcus jannaschii* DSM 2661.

**
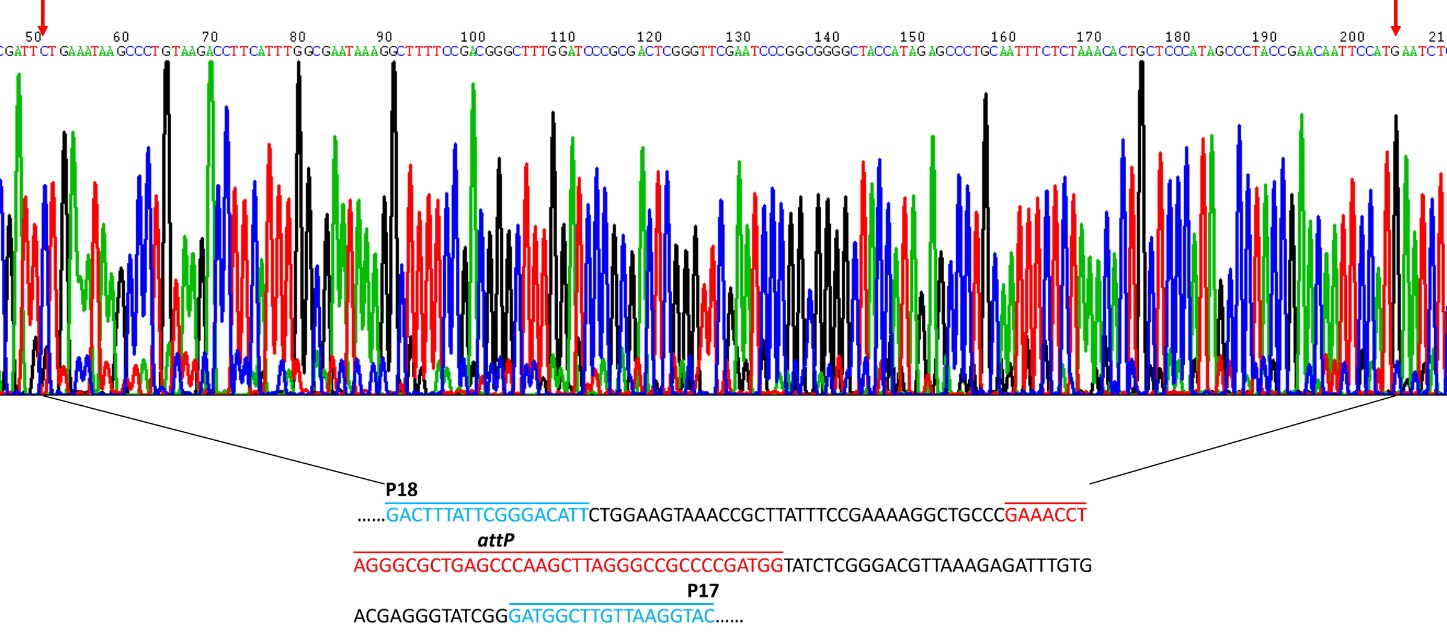
**

**Supplementary Figure 3.** Primers P17 and P18 were used to detect the episomal ring form of PYG1. A 155-bp PCR product was acquired and sequenced. The primers are displayed in blue, and the *attP* site is displayed in red.

**
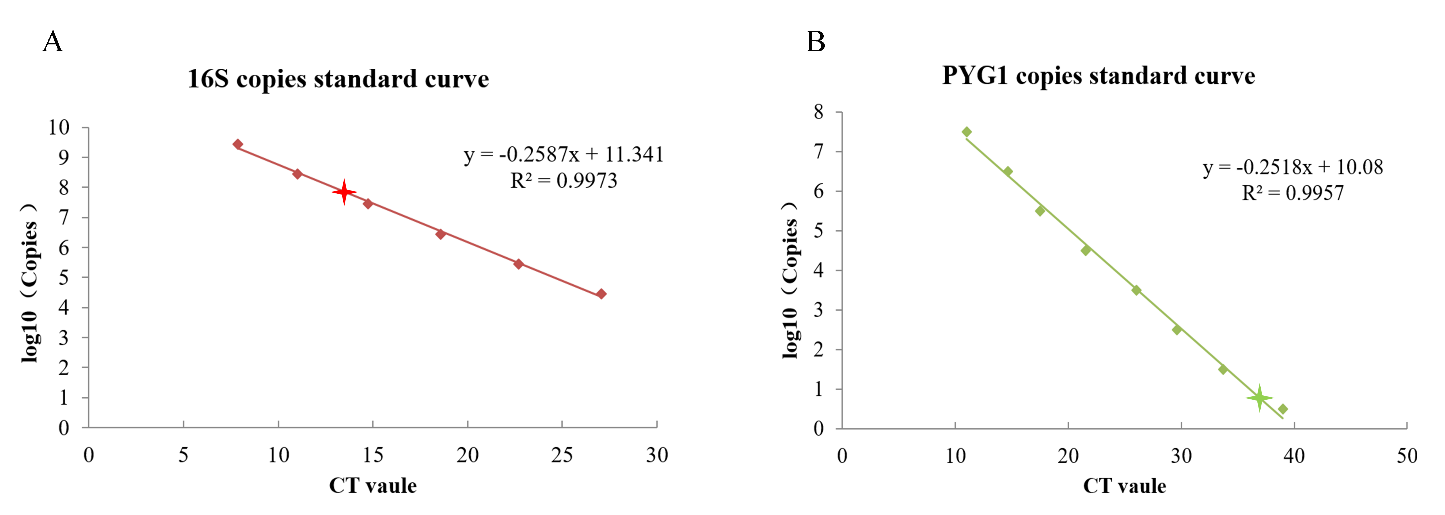
**

**Supplementary Figure 4.** Standard curves of 16S rRNA (A) and cyclized PYG1 (B). The horizontal axis is the Ct value and the longitudinal axis is the log10 value of the targets gene copies. The red star indicates the copies of the A1 16S rRNA gene. The green star indicates the copies of the A1 cyclized PYG1.

**
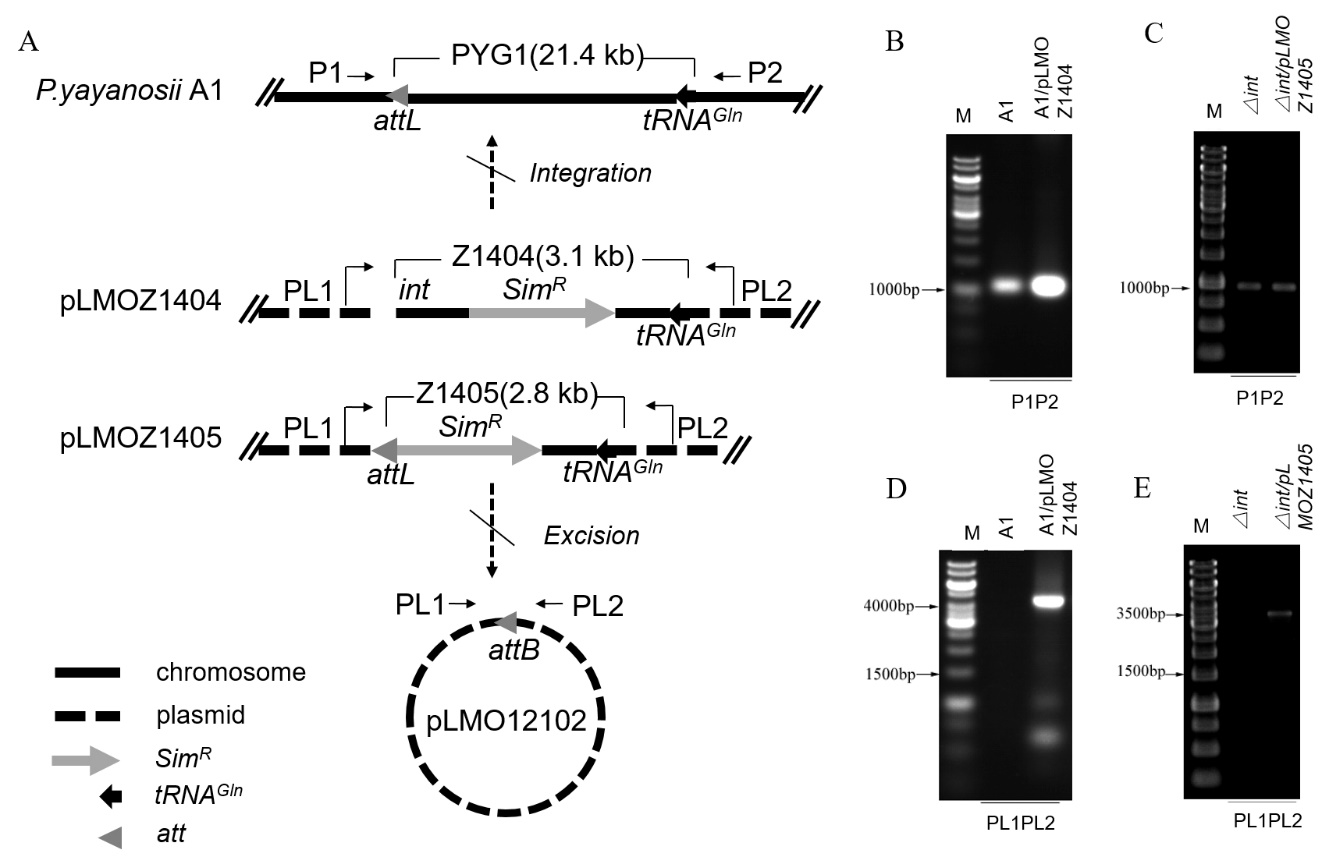
**

**Supplementary Figure 5.** The *int* gene mediates the integration and excision of mini-islands in *P. yayanosii* A1 at the *att* site. (A) A schematic of the mini-islands Z1404 and Z1405 in *P. yayanosii* A1 or the mutant strain *△int*. The mini-islands Z1404 (lacking the *att* sequence) and Z1405 (lacking the putative *int* gene *PYCH_15110*) could not be excised from plasmid pLMOZ1404/5 and integrated into *P. yayanosii* A1 or *△int* at the *attL* site (*attL* and *attB* are shown as gray triangles). (B, C) PCR analysis of the total DNA from *P. yayanosii* A1 or *△int* transformants using primers P1 and P2 detected a 1,018-bp fragment, suggesting that the mini-island Z1404/5 could not integrate into the chromosome. A PCR product of 1,018-bp was amplified in the mutant strain *△int*, suggesting that PYG1 could also excise from the chromosome of *△int*. (D, E) PCR analysis of the total DNA from *P. yayanosii* A1 or *△int* transformants using primers PL1 and PL2 detected a product of approximately 4 kb, suggesting that the mini-island Z1404/5 could not excise from plasmid pLMOZ1404/5.

## 1.2 Supplementary Table

**Table S1. Primers used in this study**

| Primers | Sequence ^a^(5’-3’) |
| --- | --- |
| Integration and excision confirmation | |
| P1 | CCGAAAGGCTCTTCACGAT |
| P2 | CCCCAGCGGTCTGTTATCTT |
| P17 | CATGGAATTGTTCGGTAG |
| P18 | CTGAAATAAGCCCTGTAA |
| P20 | CGTGAACCATCACCCAAATC |
| P21 | GATATTTACACCCGGAATAGG |
| P22 | CAATAATGAAAGAAGCATAGGC |
| PL1 | TTAAGGAGCGTGGTTTCGAGT |
| PL2 | GGAGCCTATGGAAAAACGCC |
| q16S F | GCCGATTAGGTAGTTGGTGG |
| q16S R | CCGTGTCTCAGTGTCCATC |
| q1522 F | TTGCCTCCGTGAGAAGAC |
| q1522 R | TGCAGTAAAGGAACGCCT |
| q1523 F | ATAATAGGTGGCTTTTTGCTTTG |
| q1523 R | TGATAAGTTACAAACTTCAGCCG |
| q1524 F | AAGGAGCTCGCTCACTATG |
| q1524 R | TCCTCAGGAAAGCCCAG |
| q1525 F | CTGGATTGAGGGTTACGACC |
| q1525 R | CACTTCCATGCGGTTAATCC |
| q1526 F | AGCCACGTTAGATACGCCAC |
| q1526 R | TCAACACCGTCAAGCACTCC |
| q1527 F | CGACTGTTGGAGGAATAAATCC |
| q1527 R | TGCTAAGGCTGGTCTGTTCTCT |
| Constriction of the mini-islands | |
| *Sim^R^*-F | CATCGATTTGAAAATGGAGTGAGCTGAG (*Cla* I) |
| *Sim^R^*-R | CCATCGATTTAGCGCCCCAGCATCTTGTGAGC (*Cla* I*)* |
| pLMOZ1402-aF | GGGGTACCTAATAATCGACCTGAGGAGGA (*Kpn* I) |
| pLMOZ1402-aR | ATCGATGGATCTCTGCTTCATGATTCA (*Cla* I) |
| pLMOZ1402-bF | **CAGAGATCC**ATCGATGAAGCAAATAATAATGAAGGTG (*Cla* I) |
| pLMOZ1402-bR | GGGGTACCACTTTCCTAACCTCTCGACG (*Kpn* I) |
| pLMOZ1404-F | GGGGTACCGTCGGAAAAGCCTTTATTCG (*Kpn* I) |
| pLMOZ1404-R | The sequence of pLMOZ1404-R is consistent with pLMOZ1401-bR. |
| pLMOZ1405-aF | The sequence of pLMOZ1405-aF is consistent with pLMOZ1401-aF. |
| pLMOZ1405-aR | ATCGATTTTACCACTACTGTGGAAAACAT (*Cla* I) |
| pLMOZ1405-bF | **GTAGTGGTAAA**ATCGATGAAGCAAATAATAATGAAGGTG (*Cla* I) |
| pLMOZ1405-bR | The sequence of pLMOZ1405-bR is consistent with pLMOZ1401-bR. |
| Constriction of the PYCH_15110 gene knock out plasmid | |
| *△*1511-N-F | GAAGATCTAAGCTCCTTACCTCCAGTGT (*Bgl* II) |
| *△*1511-N-R | **CTGAACCTCGTA**ATCGATTTTACCACTACTGTGGAAAACA (*Cla* I) |
| *△*1511-C-F | ATCGATTACGAGGTTCAGGAGTTGCT (*Cla* I) |
| *△*1511-C-R | GAAGATCTCTTTGGCAAGATTGGTGAGC (*Bgl* II) |
| Constriction of the PYG1 knock out plasmid | |
| *△*PYG-N-F | GAAGATCTTAATAATCGACCTGAGGAGGA (*Bgl* II) |
| *△*PYG-N-R | ATCGATGGATCTCTGCTTCATGATTCA (*Cla* I) |
| *△*PYG-C-F | **CAGAGATCC**ATCGATGAAGCAAATAATAATGAAGGTG (*Cla* I) |
| *△*PYG-C-R | GAAGATCTACTTTCCTAACCTCTCGACG (*Bgl* II) |

^a^ Restriction sites are underlined, and overlap regions are in bold.

**Table S2. Specific growth rates of *P. yayanosii* A1, A2 and △PYG1 under different growth conditions**

| **Groups ^b^** | **Specific growth rates (h^-1^) ^a^** | | |  |
| --- | --- | --- | --- | --- |
|  | ***P. yayanosii* A1** | ***P. yayanosii* A2** | **△PYG1** |  |
| A | 0.3881±0.0104 | 0.3972±0.0072 | 0.3207±0.0197 |  |
| B | 0.3880±0.0050 | 0.3758±0.0152 | 0.3094±0.0066 |  |
| C | 0.2861±0.0153 | 0.2323±0.0067 | 0.3675±0.0125 |  |
| D | 0.2969±0.0094 | 0.2730±0.0126 | 0.2994±0.0130 |  |
| E | 0.3110±0.0170 | 0.3078±0.0073 | 0.3159±0.0069 |  |
| F | 0.1514±0.0169 | 0.1662±0.0043 | 0.1665±0.0101 |  |
| G | 0.3363±0.0189 | 0.3585±0.0351 | 0.3775±0.0165 |  |
| H | 0.2921±0.0083 | 0.2847±0.0097 | 0.2712±0.0258 | |
| I | 0.3899±0.0011 | 0.3581±0.0433 | 0.3047±0.0151 | |

^a^ Specific growth rate (μ) = (lnN_t1_-lnN_to)_/ (t1-t0). The values were displayed by Mean±SD.

^b^ (A) 0.1 MPa, 95 °C, salinity 3%, pH 7; (B) 52 MPa, 95 °C, salinity 3%, pH 7; (C) 80 MPa, 95 °C, salinity 3%, pH 7; (D) 0.1 MPa, 95 °C, salinity 1.5%, pH 7; (E) 0.1 MPa, 95 °C, salinity 4%, pH 7; (F) 0.1 MPa, 95 °C, salinity 3%, pH 5.8; (G) 0.1 MPa, 95 °C, salinity 3%, pH 8.2; (H) 0.1 MPa, 90 °C, salinity 3%, pH 7; (I) 0.1 MPa, 100 °C, salinity 3%, pH 7.

## Figure Legends in Supplementary

### Figure S1. IslandViewer analyses of the *P. yayanosii* chromosome, with colored regions indicating GIs. Red, blue and orange, respectively, represent the results predicted by the Integrated, SIGI-HMM and IslandPath-DIMOB prediction methods. The genomic island PYG1 was marked with a red line.

### Figure S2. Maps of a putative functional gene cluster consisting of six genes in PYG1 that shares high identity with a plasmid of *T. barophilus* MP. Homologous genes from *T. barophilus* MP are located on plasmid pTBMP1. All of the other genes are from the chromosomes of *P. abyssi* GE5 and *P. furiosus* DSM 3638 and *Methanotorris igneus* Kol 5, *Methanocaldococcus sp.* FS406-22 and *Methanocaldococcus jannaschii* DSM 2661.

### Figure S3. Primers P17 and P18 were used to detect the episomal ring form of PYG1. A 155-bp PCR product was acquired and sequenced. The primers are displayed in blue, and the *attP* site is displayed in red.

### Figure S4. Standard curves of 16S rRNA (A) and cyclized PYG1 (B). The horizontal axis is the Ct value and the longitudinal axis is the log10 value of the targets gene copies. The red star indicates the copies of the A1 16S rRNA gene. The green star indicates the copies of the A1 cyclized PYG1.

### Figure S5. The *int* gene mediates the integration and excision of mini-islands in *P. yayanosii* A1 at the *att* site. (A) A schematic of the mini-islands Z1404 and Z1405 in *P. yayanosii* A1 or the mutant strain *△int*. The mini-islands Z1404 (lacking the *att* sequence) and Z1405 (lacking the putative *int* gene *PYCH_15110*) could not be excised from plasmid pLMOZ1404/5 and integrated into *P. yayanosii* A1 or *△int* at the *attL* site (*attL* and *attB* are shown as gray triangles). (B, C) PCR analysis of the total DNA from *P. yayanosii* A1 or *△int* transformants using primers P1 and P2 detected a 1,018-bp fragment, suggesting that the mini-island Z1404/5 could not integrate into the chromosome. A PCR product of 1,018-bp was amplified in the mutant strain *△int*, suggesting that PYG1 could also excise from the chromosome of *△int*. (D, E) PCR analysis of the total DNA from *P. yayanosii* A1 or *△int* transformants using primers PL1 and PL2 detected a product of approximately 4 kb, suggesting that the mini-island Z1404/5 could not excise from plasmid pLMOZ1404/5.

### Table S1. Primers used in the present study.

a. Restriction sites are underlined, and overlapping regions are in bold.

### Table S2. Specific growth rates of *P. yayanosii* A1, A2 and △PYG1 under different growth conditions

a. Specific growth rate (μ) = (lnN_t1_-lnN_to)_/ (t1-t0). The values were displayed by Mean±SD.

b. (A) 0.1 MPa, 95 °C, salinity 3%, pH 7; (B) 52 MPa, 95 °C, salinity 3%, pH 7; (C) 80 MPa, 95 °C, salinity 3%, pH 7; (D) 0.1 MPa, 95 °C, salinity 1.5%, pH 7; (E) 0.1 MPa, 95 °C, salinity 4%, pH 7; (F) 0.1 MPa, 95 °C, salinity 3%, pH 5.8; (G) 0.1 MPa, 95 °C, salinity 3%, pH 8.2; (H) 0.1 MPa, 90 °C, salinity 3%, pH 7; (I) 0.1 MPa, 100 °C, salinity 3%, pH 7.
