# Supplementary figures and images for: An Integrative Genomic Island Affects the Adaptations of the Piezophilic Hyperthermophilic Archaeon Pyrococcus yayanosii to High Temperature and High Hydrostatic Pressure
Source: Front Microbiol. 2016 Nov 29;7:1927. doi: 10.3389/fmicb.2016.01927 (PMC5126054; doi:10.3389/fmicb.2016.01927)

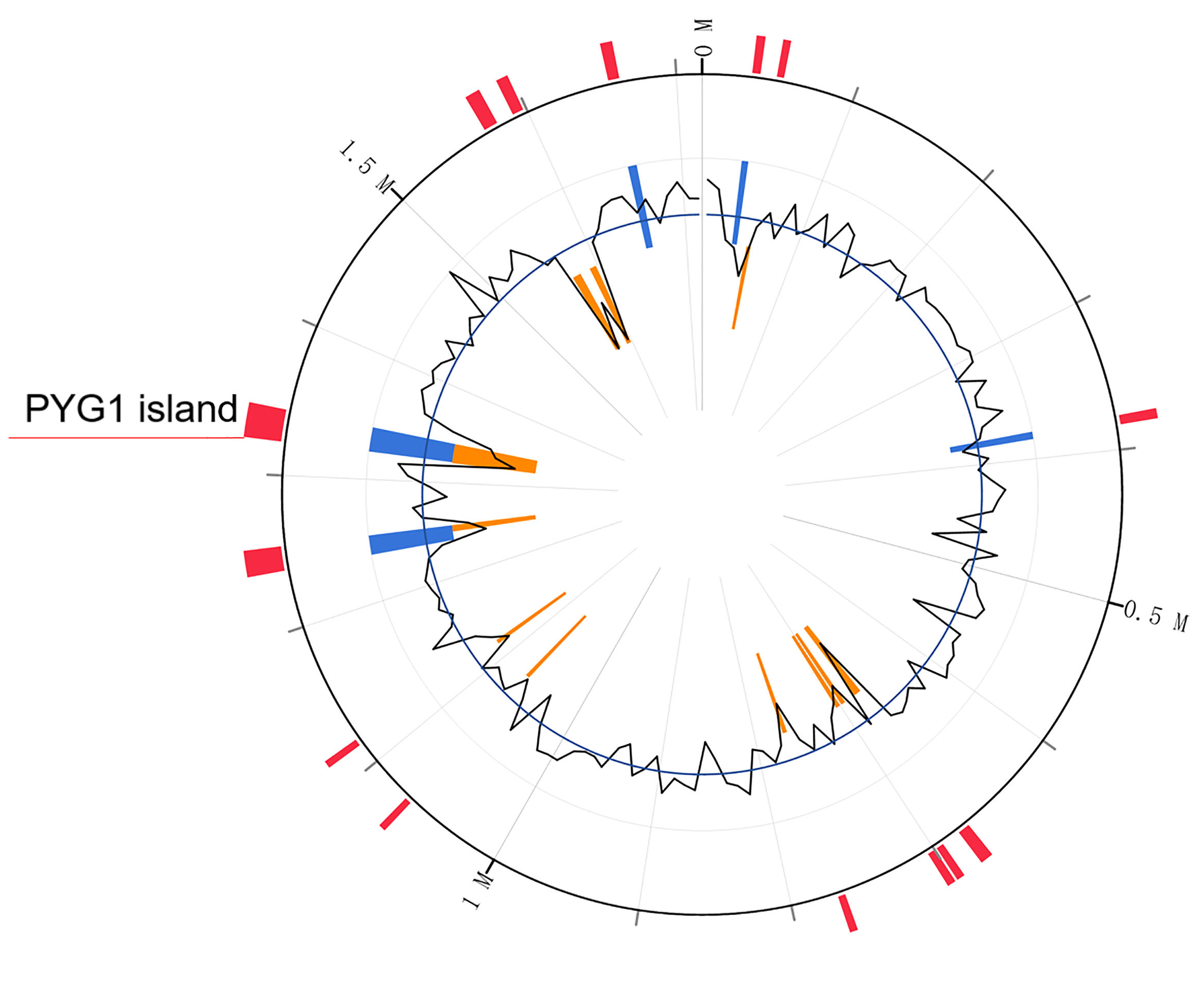

Supplement: Supplementary file 2 [file Image_1.JPEG]

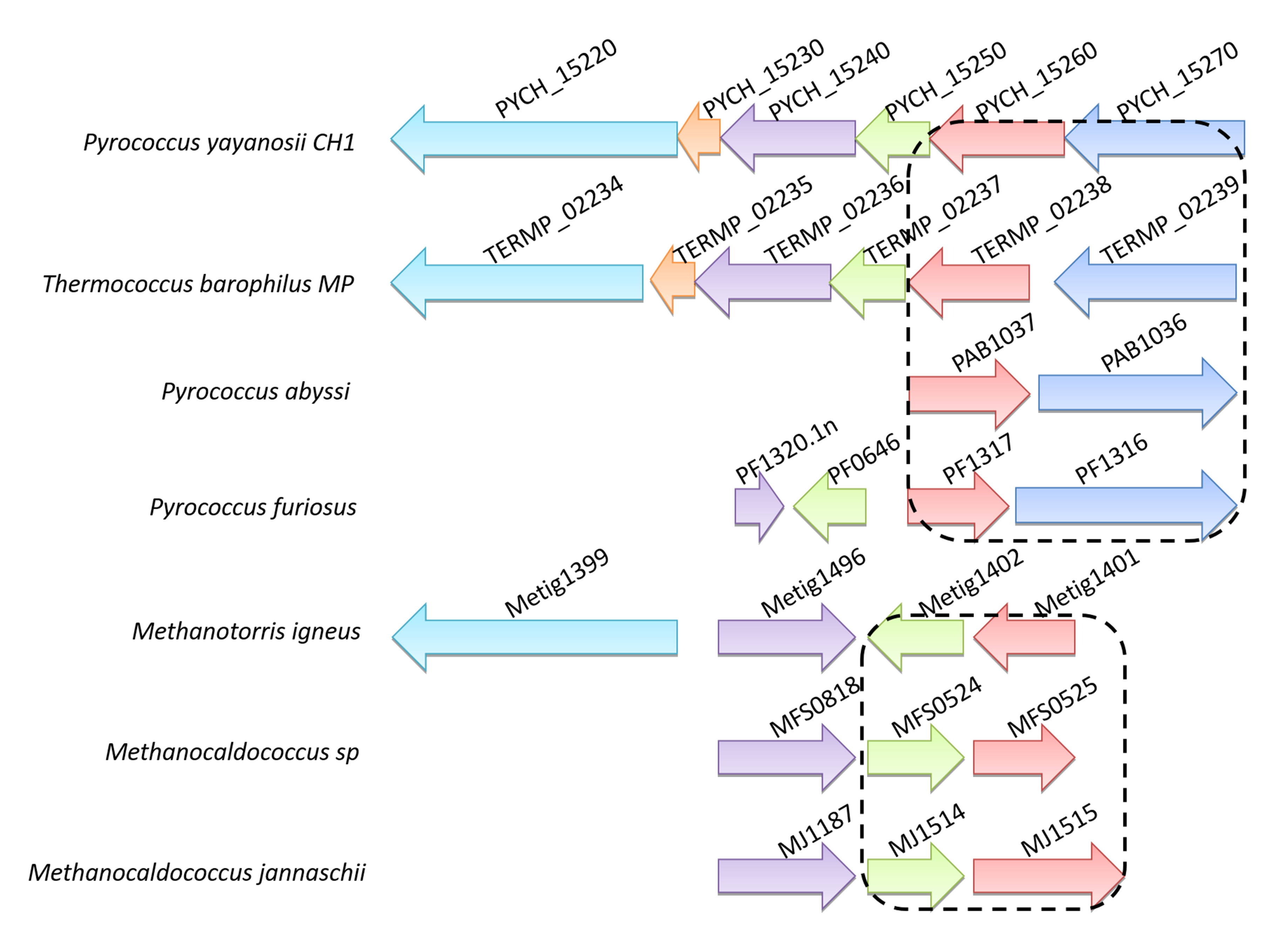

Supplement: Supplementary file 3 [file Image_2.JPEG]

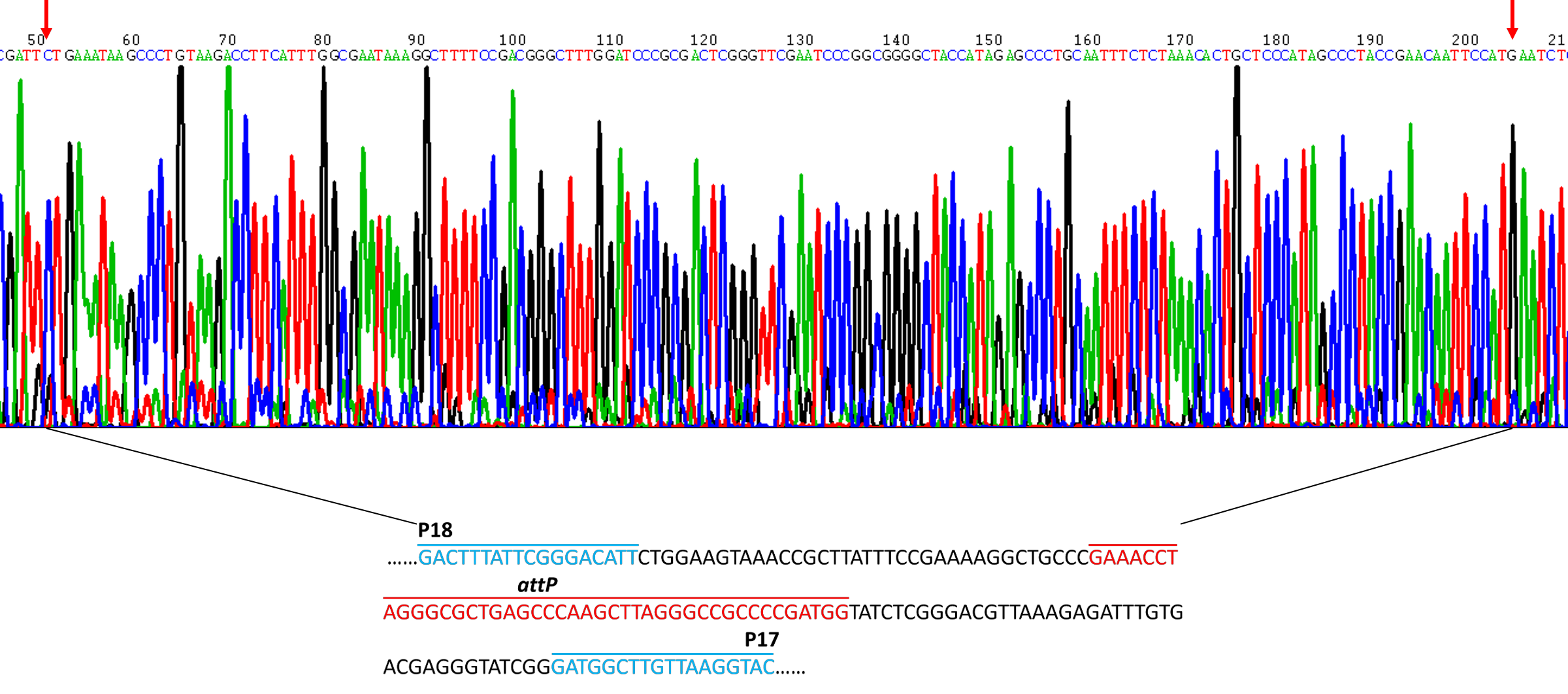

Supplement: Supplementary file 4 [file Image_3.TIF]

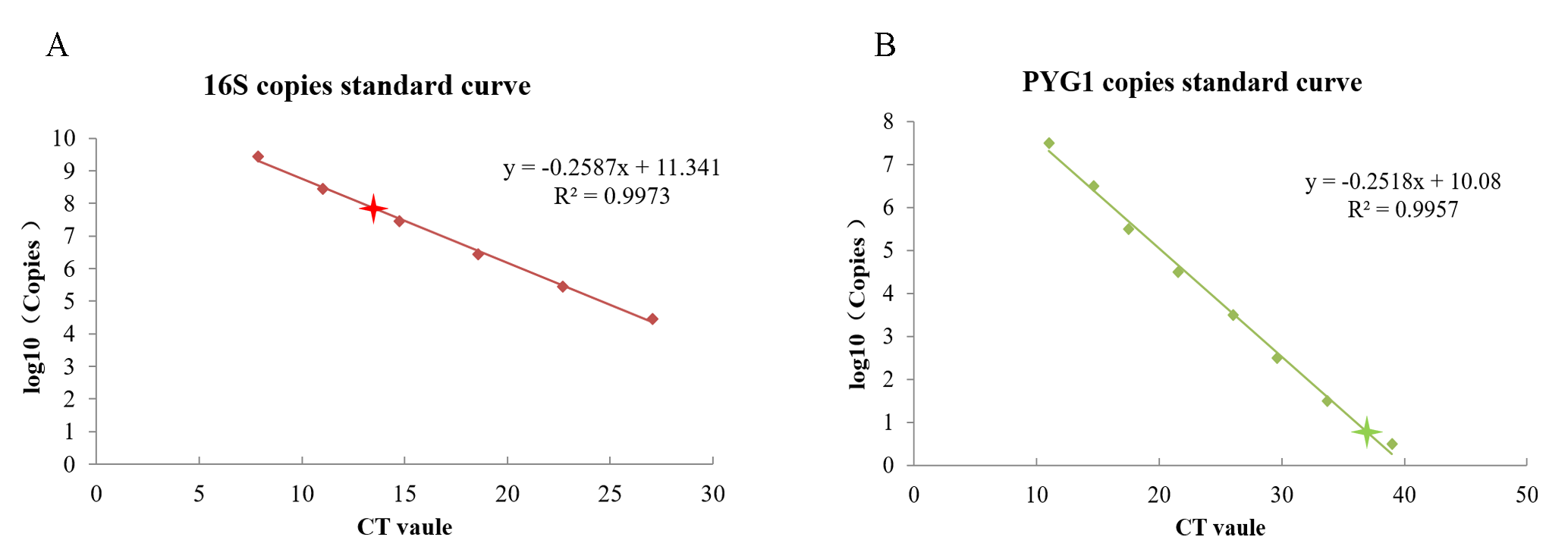

Supplement: Supplementary file 5 [file Image_4.TIF]

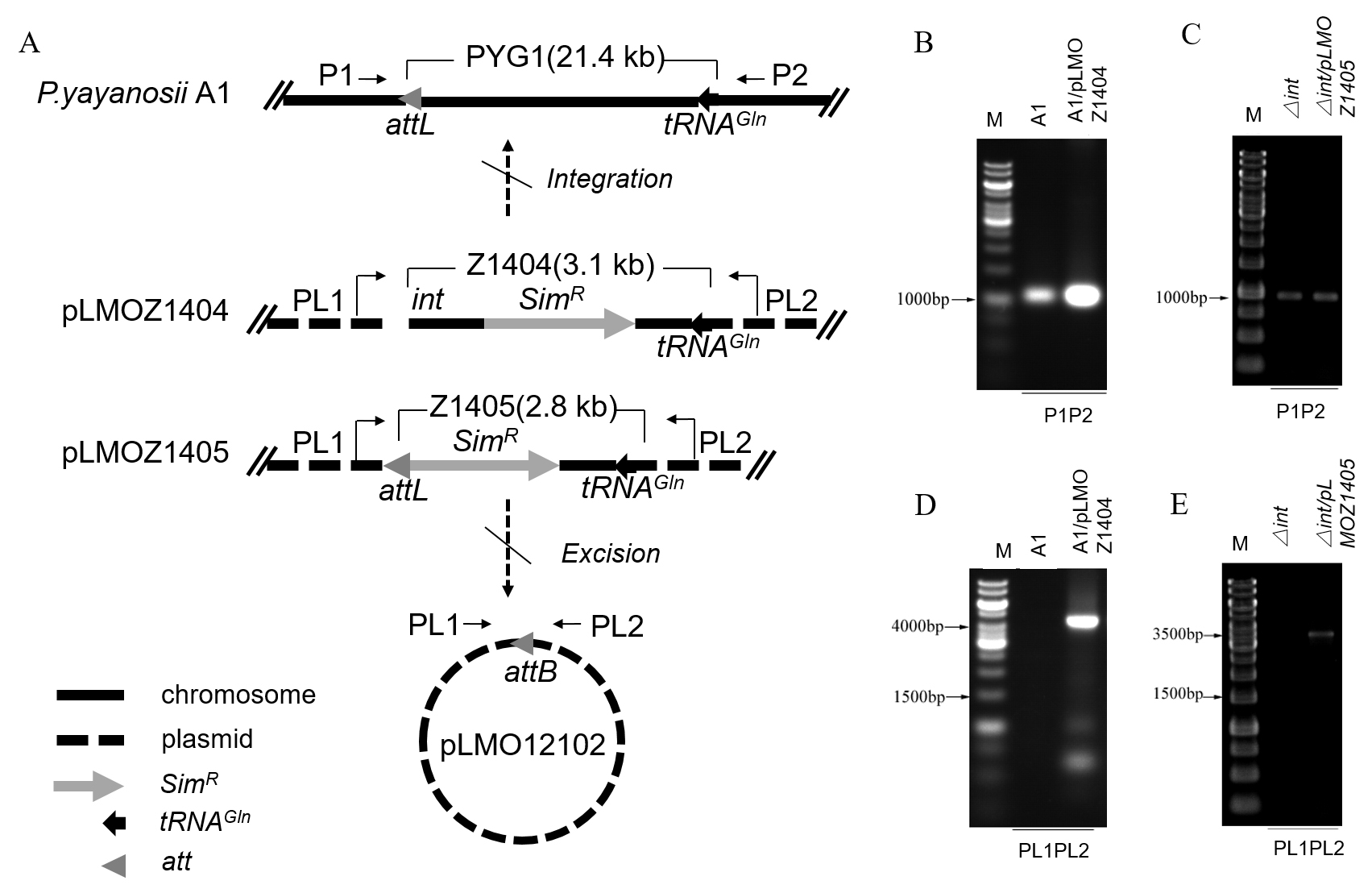

Supplement: Supplementary file 6 [file Image_5.JPEG]
